# CIRBP Enhances the Function of Yak Cumulus Cells by Activating AMPK/mTOR-Mediated Mitophagy

**DOI:** 10.3390/biom15060759

**Published:** 2025-05-24

**Authors:** Rui Zhang, Yan Cui, Yangyang Pan, Meng Wang, Sijiu Yu, Ruihua Xu, Wenbin Ma, Junqian Wang, Donglan Zhong, Zhengxing Jiao

**Affiliations:** 1 College of Veterinary Medicine, Gansu Agricultural University, Lanzhou 730000, China; 107332001040@st.gsau.edu.cn (R.Z.); cuiyan@gsau.edu.cn (Y.C.); wangmeng@gsau.edu.cn (M.W.); 1073323010063@st.gsau.edu.cn (R.X.); 107332114004@st.gsau.edu.cn (W.M.); 107332214006@st.gsau.edu.cn (J.W.); 107332214009@st.gsau.edu.cn (D.Z.); 1073323140007@st.gsau.edu.cn (Z.J.); 2 Gansu Livestock Embryo Engineering Technology Innovation Center, Lanzhou 730000, China

**Keywords:** CIRBP, mitophagy, apoptosis, cumulus diffusion, ovarian steroid hormones

## Abstract

Cold-inducible RNA-binding protein (CIRBP) has been reported to be involved in various cellular functions by regulating programmed cell death (PCD). However, the specific mechanism and function of CIRBP in regulating mitochondrial autophagy are still unclear. In this study, we found that CIRBP induced mitophagy through the AMPK/mTOR pathway to improve the function of yak cumulus cells (YCCs). We observed that low temperatures (32 °C) activated autophagy, increased E2 and P4 secretion, and up-regulated CIRBP expression. CIRBP overexpression activated mitophagy in YCCs, promoted cumulus diffusion, enhanced E2 and P4 synthesis and secretion, and inhibited apoptosis. CIRBP overexpression significantly attenuated the dysfunction of YCCs induced by the inhibition of mitophagy, whereas the activation of mitophagy exerted the same effect as CIRBP overexpression. DOX HCL is an AMPK/mTOR pathway inhibitor. CIRBP overexpression can successfully alleviate the inhibition of mitophagy caused by DOX HCL inhibiting the AMPK/mTOR pathway and can significantly enhance the mitophagy induced by AMPK/mTOR pathway activation in YCCs. Furthermore, we found that the increased expression of CIRBP protein alleviated the apoptosis caused by AKT pathway activation. In summary, CIRBP promoted mitophagy by activating AMPK/mTOR pathway, thereby promoting the synthesis and secretion of steroid hormones and cumulus diffusion in YCCs and enhancing YCCs survival through activating autophagy and AKT signaling pathway, and then improve the function of YCCs. Our research provided new perspectives on CIRBP’s regulation of cell death and highlighted its potential role in female reproductive systems.

## 1. Introduction

As a protein that reacts to various environmental stressors, Cold-inducible RNA-binding protein (CIRBP) boosts cellular resistance to conditions like hypothermia, lack of oxygen, and UV light. It was first discovered as a transcript that is activated by DNA damage [1]. CIRBP is widely expressed in multiple organs and various cells of many animals [2]. Research indicates that the CIRBP levels increase in the brains and hearts of rats subjected to chronic intermittent hypothermia [3]. At hypothermia, by binding to the CIRBP promoter, Sp1 induces CIRBP protein displacement and stimulates its expression [4]. In addition, during testicular injury, heat stress down-regulates CIRBP expression by transforming growth factor β [5]. This indicates that different cell types, cell microenvironment and cell localization all affect CIRBP transcription. As an RNA chaperone that promotes RNA translation, after the transcription of target genes, CIRBP regulates the level of target genes by binding to their mRNA. Subsequently, CIRBP is involved in the regulation of a variety of signaling pathways associated with cell proliferation and reproductive development [6,7,8]. CIRBP plays an important role in male mammalian reproductive physiology by binding to infertility-related mRNA in the testis and maintaining its gene stability or regulating pathways related to spermatogenesis. For example, down-regulation of CIRBP activates key pathways that affect spermatogenesis, such as SAPK/JNK MAPK and p44/p42 [9]. Dryk1b/Mirk is a key enzyme involved in the regulation of cell growth arrest, and CIRBP promotes spermatogonia differentiation by interacting with it [10]. CIRBP plays a role in oocyte and embryo cryopreservation during female reproduction. Studies have shown that CIRBP maintains the integrity and protects the function of vitrified mature oocytes in mice [11]. CIRBP is involved in regulating the process of embryo attachment, involving adhesion molecules such as β-catenin [12]. In summary, existing data suggest the potential role of CIRBP in mammalian reproductive development; however, most current research on the regulation of reproductive physiology by CIRBP is related to male animals, and its specific mechanism in female animal reproduction is unknown. Notably, a study found that CIRBP promotes ferritin deposition by activating ferritin autophagy [7]. It shows the regulatory role of CIRBP in programmed cell death (PCD).

PCD is a process in which the irreversible degradation of cell function eventually leads to the loss of cell integrity, including apoptosis, autophagy, pyroptosis and ferroptosis [13]. Apoptosis is a highly autonomous and PCD process of normal cells in the body under external stimulation. This involves complex signal cascade amplification, which eventually leads to the orderly termination of cells [14]. Existing evidence suggests that CIRBP alleviates multiorgan injury by modulating apoptosis. CIRBP reportedly alleviates acute kidney injury by inhibiting mitophagy pathway [15]. CIRBP up-regulation significantly reduces oxidative damage-induced cardiomyocyte injury and apoptosis in mice [16]. CIRBP protects rat neuronal cells from amyloid β toxic damage by anti-apoptotic via Akt and ERK pathways [17]. In contrast, some researchers have found that CIRBP released in shock can directly activate lung endothelial cells (ECs) pyroptosis, leading to lung injury [18]. High CIRBP levels increase oxidative stress and apoptosis in rat hepatocytes, which aggravates hepatic ischemia-reperfusion injury [19]. These data indicate that the regulation of apoptosis by CIRBP is dynamic, which may be related to the cell type and the conditions that induce its expression. Autophagy is another mechanism of PCD that occurs in eukaryotes under various physiological and pathological conditions. Its primary function is to decompose unwanted components into energy and essential materials [20]. Studies have shown that CIRBP activates ferritin autophagy to promote ferroptosis [7]. Another study showed that CIRBP mediates TLR4 signaling to induce mitophagy and regulate macrophage death after trauma [21]. Although these data indicate that CIRBP can participate in the regulation of a variety of cellular physiological and pathological processes by regulating programmed cell death. However, the molecular mechanism of its regulation of programmed cell death is still unclear, and research on yak cell death has not been reported.

A recent study identified CIRBP and the autophagy-related factors LC3B and P62 as direct or indirect regulators of cholesterol efflux and phagocytosis [22]. Steroid hormones are derivatives of cholesterol [23]. This seems to suggest a potential regulatory relationship between CIRBP and autophagy on steroid hormone synthesis.

Autophagy has been shown to regulate steroid synthesis and secretion. For example, activation of autophagy in porcine Leydig cells can alleviate the decrease in testosterone secretion caused by diethyl hexyl phthalate (DEHP) exposure through m6A epigenetic regulation [24]. Further, inhibition of autophagy leads to a decrease in the expression of the estradiol-synthesis-related factor CYP19A1, thereby reducing estradiol secretion [25]. Granulosa cells (GCs) provide nutrition and microenvironment for oocyte development, and the expansion degree of cumulus cells plays an important role in oocyte maturation. Previous studies have shown that HIF-1α can promote the expression of diffusion factors in yak cumulus cells by activating autophagy, affecting yak oocyte maturation and early embryonic development [26]. However, the underlying mechanism of PCD involved in cumulus diffusion and steroid hormone synthesis and secretion regulation remains to be determined.

Yaks are typical plateau mammals that inhabit cold hypoxic environments, and their low reproduction rate restricts the development of the yak industry [27]. Yak cumulus cells provide the developmental microenvironment for oocytes, and normal oocyte function is necessary for oocyte development. In summary, the purpose of this study was to investigate the effects of CIRBP regulation of mitophagy on the function and apoptosis of yak cumulus cells, and to study the mechanism of CIRBP regulation of mitophagy and apoptosis. The results provide new evidence for exploring the mechanism of CIRBP regulating PCD and provide basic data for CIRBP participating in the regulation of mammalian reproductive physiology.

## 2. Materials and Methods

### 2.1. Culture of Primary Yak Cumulus Cells (YCCs)

Ovarian samples were collected from a slaughtering company in Xining City, Qinghai Province, in line with the national animal welfare protocols. Collection of follicular stage ovaries from adult yaks was carried out. The ovaries were washed with normal saline containing penicillium streptomycin at 37 °C until there was no obvious blood and odor. Cumulus–oocyte complex (COC) in follicular fluid was collected and cultured in maturation medium. Upon the emergence of the first polar body, a marker of COC maturation, the CCs and oocytes were digested with hyaluronidase. After separation, the oocytes were discarded, and the CCs cells were collected and made into a suspension, which was cultured at 5% CO_2_ and 37 °C.

### 2.2. Low-Temperature Treatment and Transfection of YCCs

When the well-grown YCCs reached an appropriate degree of fusion, the cells were treatment to a low temperature (32 °C), and the samples were collected after 6 and 12 h of low-temperature culture. pGCMV/MCS/Neo-CIRBP is abbreviated as PEX-CIRBP as the CIRBP overexpression group, and pGCMV/MCS/Neo (PEX3) is abbreviated as PEX-NC as the negative control group (the vectors were all constructed by Shanghai Jima Co., Shanghai, China); at the same time, a blank control group, ‘control’, was set up to clarify the differences between the treatment group, the control group and the negative control group. According to the instructions of the transfection reagent, Lipo8000^TM^ Transfection Reagent (Lipo8000, Beyotime Biotechnology, C0533, Shanghai, China), the plasmid DNA (μg) and Lipo8000 (μL) should be mixed in a ratio of 1:1.6 to prepare the transfection complex. PEX-CIRBP or PEX-NC should be transfected into YCCs cells under conditions of 37 °C and 5% O2 for 24 h or 48 h. In this experiment, the transfection efficiency at 24 h was approximately 60%, while at 48 h, it was approximately 80%.

### 2.3. Drug Treatment of YCCs

The drugs were diluted according to the manufacturer’s instructions for 3-Methyladenine (3-MA), Rapamycin (RAPA), Acadesine (AICAR), Doxorubicin (DOX) HCL, LY294002, and 740 Y-P. After 24 h of transfection, the drug treatment group (3MA treatment for 12 h) was added, and samples were collected after 24 h of culture. Drugs 3-MA, RAPA, AICAR, DOX HCL, LY294002 and 740 Y-P were purchased from China MCE. YCCs were treated with 3MA to 7.5 mM/mL as an autophagy inhibition group. YCCs were treated with RAPA to 15 μM/mL as an autophagy activation group. YCCs were treated with 740 Y-P diluted to 20 μM/mL as a PI3K/AKT activation group. YCCs were treated with LY294002 to 30 μM/mL as a PI3K/AKT inhibition group. YCCs were treated with AICAR diluted to 500 μM/mL as an AMPK/mTOR activation group or mitophagy activation group. YCCs were treated with DOX HCL to 200 μM/mL as an AMPK/mTOR inhibition group or mitochondrial inhibition group.

### 2.4. Immunofluorescence Staining

YCCs with good morphology were cultured on coverslips. When the degree of cell fusion reached 70%, paraformaldehyde fixation, Triton X-100 solution permeabilization, blocking in 2% BSA solution, primary antibody incubation overnight, and DAPI staining of nuclei after secondary antibody staining were performed. Final protein localization was carried out using laser confocal microscopy. The antibodies and dilution ratios used in this experiment are as follows: CIRBP (Affinity, DF2643, Liyang, China, 1:200), COX2 (Affinity, AF7003, Liyang, China, 1:200), PTX3 (Affinity, DF3132, Liyang, China, 1:200), TSG6 (Affinity, AF5492, Liyang, China, 1:200), HAS2 (Affinity, BF0034, Liyang, China, 1:200).

### 2.5. RT-PCR

Total RNA was extracted and quantified using TransZol reagent (TransGen, Beijing, China), Evo M-MLV reagent and SYBR Green Premix Pro Taq HS qPCR reagent (Accurate Biotechnology, Changsha, China). The specific primer sequences were as follows (Table 1):

### 2.6. Detection of E2 and P4 Secretion

According to the instructions of the Bovine Estradiol ELISA kit (Shanghai Fenxi Biology, FXs01960, Shanghai, China) and the Bovine PROG ELISA kit (Shanghai Meilian Biology, E—0002921, Shanghai, China), the E2 and P4 secretion levels in the cell supernatants of the treatment group, PEX-CIRBP, 3MA, PEX + 3MA and RAPA; the negative control group, PEX-NC; and the blank control group were detected.

### 2.7. Western Blotting

Total cellular proteins were extracted according to the instructions of RIPA Tissue Lysate (Transgenic, Beijing, China). The protein was separated by SDS-PAGE, transferred to PVDF and blocked with 5% skim milk, and then specifically bound to primary antibody CIRBP (Affinity, DF2643, Liyang, China, 1:1000), LC3B (Proteintech, 14600-1-AP, Wuhan, China, 1:1500), ATG5 (Bioss, bs-4005R, Beijing, China, 1:800), Beclin-1 (Proteintech, 11306-1-AP, 1:1500), P62 (Proteintech, 18420-1-AP, Wuhan, China 1:1500), STAR (Affinity, Liyang, China, DF6192, 1:2000), CYP1A1 (Affinity, AF5312, Liyang, China, 1:1000), CYP11A1 (Affinity, DF4697, Liyang, China, 1:1000), CYP17A1 (Affinity, AF5210, Liyang, China, 1:1000), CYP19A1 (Affinity, AF5229, Liyang, China, 1:1000), COX2 (Affinity, AF7003, Liyang, China, 1:1000), PTX3 (Affinity, Liyang, China, DF3132, 1:1000), TSG6 (Affinity, AF5492, Liyang, China, 1:1000), HAS2 (Affinity, BF0034, Liyang, China, 1:1000), β-actin (Affinity, AF7018, Liyang, China, 1:8000), GAPDH (Proteintech, 10494-1-AP, Wuhan, China 1:20,000). After washing, the secondary antibody was incubated and a chemiluminescent solution (ECL Luminescent Solution, Abcam, Cambridge, UK) was added dropwise. Imaging and semi-quantitative protein expression were performed using GE AI600 imaging system and Image J 1.51j8 software.

### 2.8. Flow Cytometry

The cells were treated with drugs or plasmid transfection experiments; the cells were digested by trypsin, washed twice with buffer, and the cell precipitates were collected and resuspended, added to the antibody group and isotype control group centrifuge tubes for co-incubation in the dark at room temperature for 15 min, and then centrifuged and resuspended. Blank control, isotype control and cells stained with target antibody were detected separately on the flow-cytometry-measuring equipment (BriCyte E6 Flow Cytometer, Shenzhen mindary global, China). The blank control and isotype control parameters were set and sample data were collected according to the parameters to collect 2 × 10^4^ valid cells. The same group of samples was tested 3 times to ensure the accuracy of the data.

### 2.9. Detection of Ad-mCherry-GFP-LC3B

YCCs were labeled with Ad-mCherry-GFP-LC3B for 24 h. After treatment of the cells with reference to Method (Section 2.2), the cells were fixed with paraformaldehyde, and the nucleus was stained with DAPI and blocked with an anti-fluorescence quencher. Autophagy flux was assessed by imaging using laser confocal fluorescence microscopy.

### 2.10. Statistical Analysis

Relative expression was calculated by the 2^−ΔΔCt^ algorithm, and the statistical significance of the difference was analyzed by one-way analysis of variance (ANOVA and average paired comparison. GraphPad Prism 9.5 was used to draw the data graph. The specific data repetition number (*n* value) and the significance of the difference were marked in the graph. The results were expressed as mean ± standard error (mean ± SE), *: *p* < 0.05, **: *p* < 0.01, NS: no difference.

## 3. Results

### 3.1. Effect of Low Temperature on CIRBP Expression, Mitophagy and E2 and P4 Secretion in Cells

**Figure 1:**
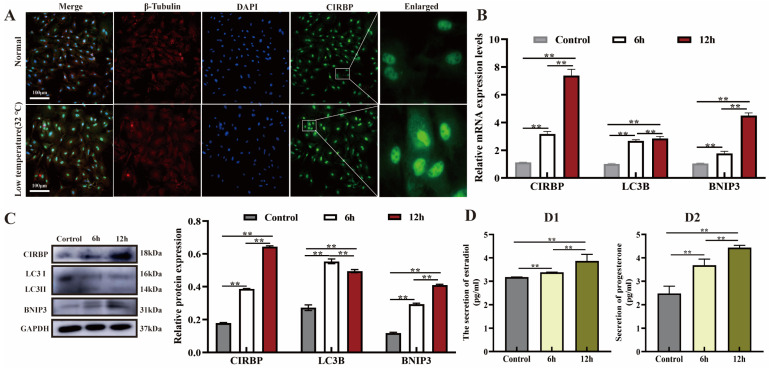
Effect of low temperature on CIRBP expression, mitophagy and E2 and P4 secretion in cells. (**A**) Subcellular localization of CIRBP in YCCs after low-temperature treatment. (**B**) Expression levels of LC3, BNIP3 and CIRBP after low-temperature treatment detected by qRT-PCR, *n* = 4. (**C**) Expression levels of LC3, BNIP3 and CIRBP after low-temperature treatment detected by WB, *n* = 3. (**D**) Secretion of E2 (**D1**) and P4 (**D2**) after low-temperature treatment, *n* = 4t. Values are mean ± SE, **: *p* < 0.01. Original Western blot images are provided in the Supplementary Materials.

CIRBP is up-regulated in response to low temperature and its protein position undergoes migratory changes to play an important role [28]. We observed the subcellular localization of CIRBP after low-temperature treatment of YCCs for 6 h and 12 h and evaluated mitophagy, E2 and P4 secretion, and CIRBP expression. The results showed that low-temperature treatment for 12 h significantly up-regulated the expression of CIRBP in YCCs and resulted in the migration of CIRBP from the nucleus to the cytoplasm (Figure 1A,B). In addition, the expression levels of BNIP3 and LC3B proteins in YCCs were significantly up-regulated by low temperature, and E2 and P4 secretion increased (Figure 1C,D). These results suggest that low temperature induces the expression of CIRBP in YCCs, activates mitophagy, and increases steroid hormone secretion.

### 3.2. Overexpression of CIRBP Activates Mitophagy in YCCs

**Figure 2:**
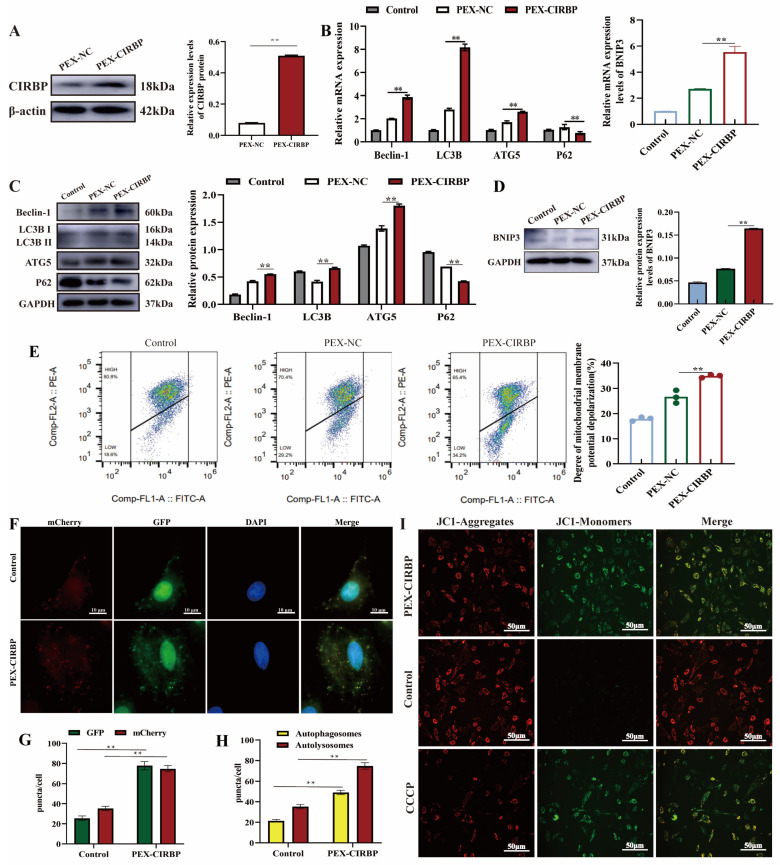
Overexpression of CIRBP activates mitophagy in YCCs. (**A**) Evaluation of CIRBP overexpression modeling in YCCs, *n* = 3. (**B**) The mRNA expression level of mitophagy-related factors, *n* = 4. (**C**,**D**) The protein expression level of mitophagy-related factors, *n* = 3. (**E**) Flow cytometry detection of mitochondrial membrane potential, *n* = 3. (**F**) Ad-mCherry-GFP-LC3B labeling assay Bar = 10 μm. (**G**) The amount of GFP-LC3B and autophagosome/autolysosome. (**H**) The amount of autophagosomes and autolysosomes. (**I**) Detection of mitochondrial membrane potential by JC-I staining. Values are mean ± SE, **: *p* < 0.01. Original Western blot images are provided in the Supplementary Materials.

Mitophagy controls the quantity and quality of intracellular mitochondria and is essential for maintaining cell survival. We previously found that CIRBP regulates autophagy. In order to illustrate the relationship between CIRBP and mitophagy induced by low temperature, we established a CIRBP overexpression model to evaluate the regulatory effect of CIRBP on YCC mitophagy. The data showed that CIRBP overexpression significantly up-regulated the ratio of LC3 I/II, induced the expression of BNIP3, Beclin-1 and ATG5 proteins, and inhibited P62 (Figure 2A–D). To further clarify the effect of CIRBP on mitophagy in YCCs, we used flow cytometry to detect the mitochondrial membrane potential and used AD-Ad-mCherry-GFP-LC3B double-label staining to detect the autophagic fluxes in YCCs after CIRBP overexpression. The results showed that the mitochondrial membrane potential of the CIRBP overexpression group was depolarized, and more red (autolysosomes) and yellow (autophagosomes) spots were observed (Figure 2E–H). In addition, JC-1 staining showed that the change in mitochondrial membrane potential after up-regulation of CIRBP was similar to that after Carbonyl cyanide 3-chlorophenylhydrazone (CCCP, a type of mitochondrial electron transport chain inhibitor, as a positive control to induce the decrease in mitochondrial membrane potential)-positive treatment, and green fluorescence was enhanced (Figure 2I). This suggested that the mitochondrial membrane potential was impaired. These results indicated that overexpression of CIRBP caused impaired depolarization of the mitochondrial membrane potential in YCCs, which, in turn, activated mitophagy.

### 3.3. Overexpression of CIRBP Improves YCC Function and Inhibits Apoptosis

**Figure 3:**
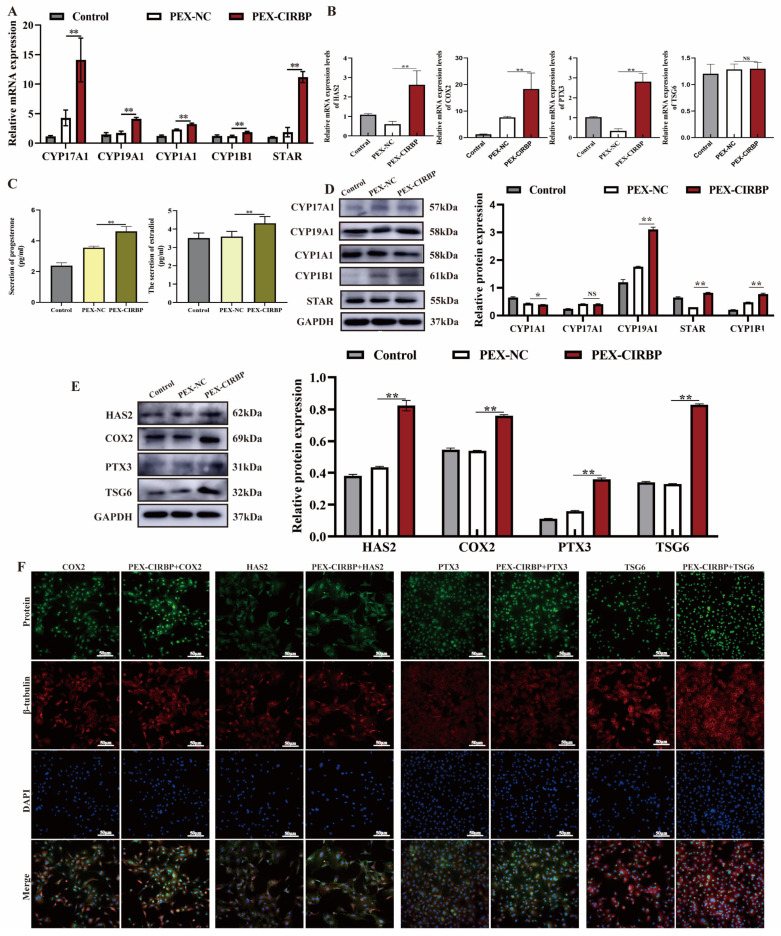
Overexpression of CIRBP improves YCC function. (**A**) Steroid-synthesis-related factors expression level after CIRBP overexpression, *n* = 4. (**B**) The mRNA expression level of cumulus-diffusion-related factors, *n* = 4. (**C**) The secretion of E2 and P4, *n* = 4. (**D**) E2 and P4 synthesis-related protein bands and analysis, *n* = 3. (**E**) Cumulus-diffusion-related protein bands and analysis, *n* = 3. (**F**) Immunofluorescence detection of cumulus-diffusion-related proteins. Values are mean ± SE, *: *p* < 0.05, **: *p* < 0.01, NS: no difference. Original Western blot images are provided in the Supplementary Materials.

CCs provide energy and a microenvironment for oocyte development. Cumulus diffusion and the secretion of ovarian steroid hormones are essential for regulating oocyte development and maturation [29]. Therefore, we explored the effects of CIRBP overexpression on the synthesis and secretion of E2 and P4 and cumulus diffusion in YCCs. qRT-PCR revealed that CIRBP overexpression significantly up-regulated the mRNA expression levels of CYP1B1, CYP17A1, CYP19A1, STAR, CYP1A1, HAS2, COX2 and PTX3, and there was no significant difference in the expression level of TSG-6 mRNA (Figure 3A,B). The ELISA results showed that overexpression of CIRBP increased the secretion of E2 and P4 (Figure 3C). In addition, overexpression of CIRBP up-regulated the synthesis of E2 and P4 and the expression of cumulus-diffusion-related proteins (Figure 3D,E). The immunofluorescence results were consistent with the WB results, and up-regulated CIRBP induced the translation of cumulus-diffusion-related proteins (Figure 3F). The results suggested that the overexpression of CIRBP improved YCC function.

**Figure 4:**
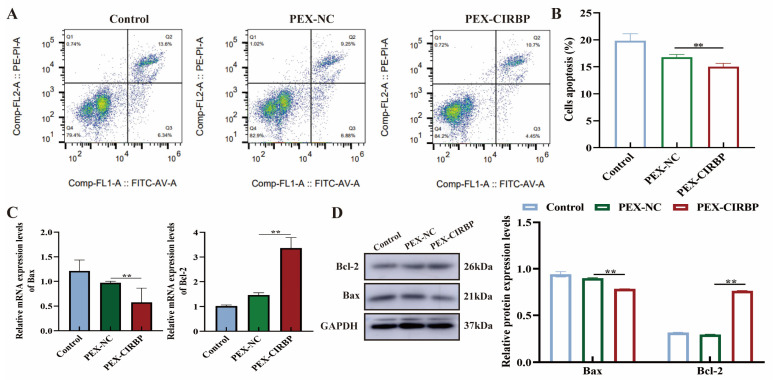
CIRBP inhibits apoptosis of YCCs. (**A**,**B**) The apoptosis level of YCCs after overexpression of CIRBP was detected, *n* = 3. (**C**) Apoptosis-related factor mRNA expression level, *n* = 4. (**D**) Apoptosis-related protein bands and analysis, *n* = 3. Values are mean ± SE, **: *p* < 0.01,. Original Western blot images are provided in the Supplementary Materials.

Previous studies have suggested that apoptosis is a mechanism leading to follicular atresia. Therefore, we further examined the effect of CIRBP on YCC apoptosis. The results showed that overexpression of CIRBP reduced the apoptosis rate. (Figure 4A,B). The expression levels of apoptosis-related proteins were consistent with the trend in mRNA expression levels. The overexpression of CIRBP significantly promoted the expression level of Bcl-2 and reduced Bax. In summary, this study found that CIRBP overexpression promoted the synthesis and secretion of E2 and P4 in YCCs, promoted cumulus diffusion, and inhibited YCC apoptosis.

### 3.4. CIRBP Enhances YCC Function and Inhibits Apoptosis by Activating Mitophagy

**Figure 5:**
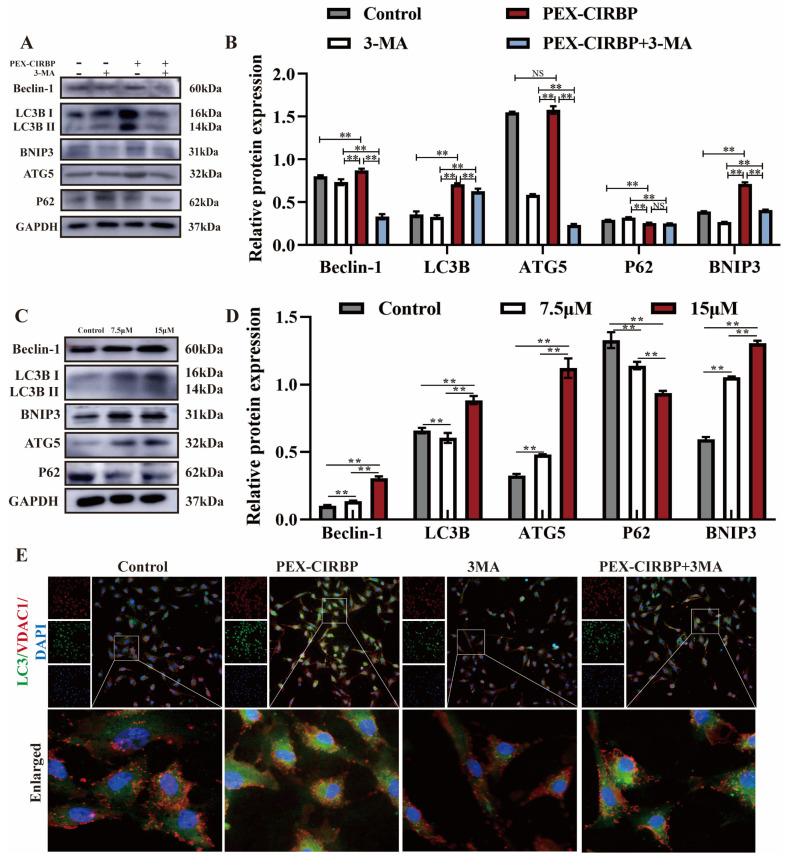
CIRBP regulates YCCs mitophagy. (**A**,**B**) Mitophagy-related protein bands and analysis after co-treatment of PEX-CIRBP and 3MA, *n* = 3. (**C**,**D**) Mitophagy-related protein bands and analysis after RAPA treatment (**E**) LC3 and VDAC1 were detected by immunofluorescence co-staining, *n* = 3. Values are mean ± SE, **: *p* < 0.01, NS: no difference. Original Western blot images are provided in the Supplementary Materials.

To determine whether CIRBP affects YCC function and apoptosis by regulating mitophagy, YCCs were treated with PEX-CIRBP and mitophagy inhibitor 3MA to detect the regulation of CIRBP on YCC mitophagy. Research has found that 3-MA treatment inhibits the expression of mitochondrial-autophagy-related proteins, while the overexpression of CIRBP alleviated the down-regulation effect of 3-MA on mitochondrial-autophagy-related proteins (Figure 5A,B). Further treatment of YCCs with the mitophagy activator RAPA showed similar results to those of CIRBP overexpression (Figure 5C,D). In addition, immunofluorescence co-staining of LC3 and the mitochondrial membrane protein VDAC1 showed that when CIRBP was overexpressed, the co-localization of LC3 and VDAC1 was enhanced. The co-treatment group could alleviate the decreased co-localization of LC3 and VDAC1 caused by 3MA. These results suggested that CIRBP was involved in the regulation of mitophagy in YCCs.

**Figure 6:**
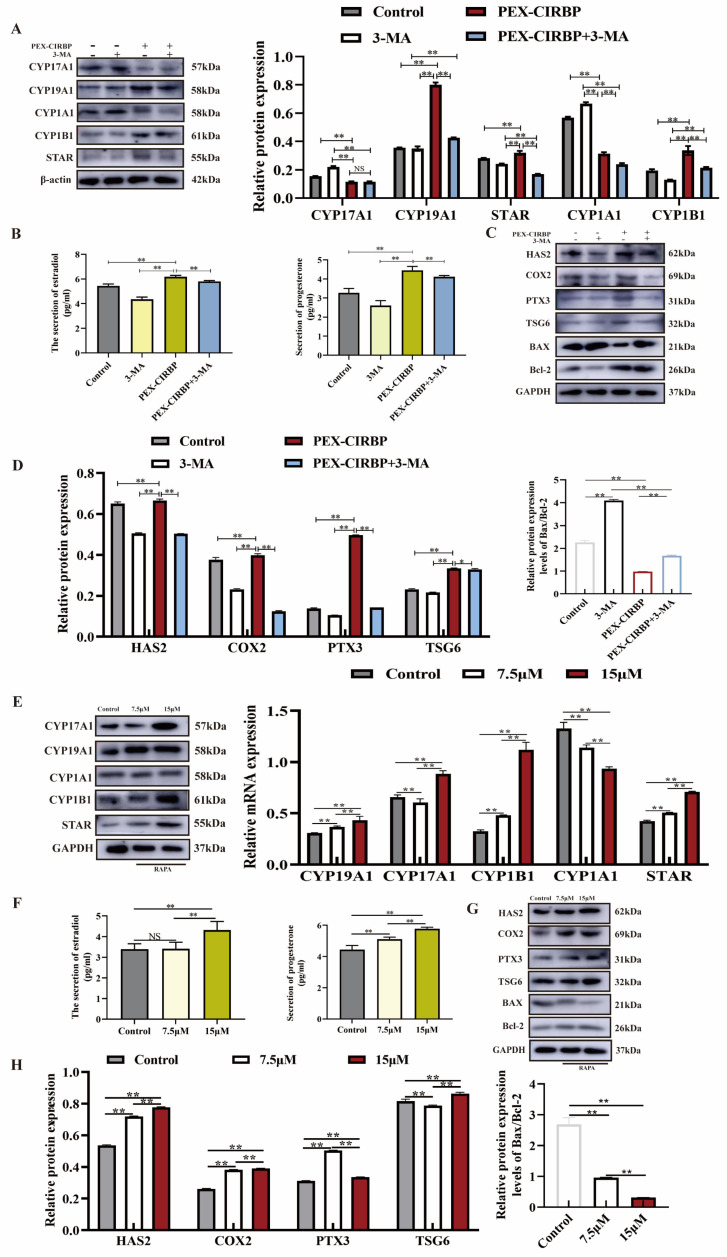
CIRBP enhances YCC function and inhibits apoptosis by activating mitophagy. (**A**–**D**) After co-treatment of PEX-CIRBP and 3MA, *n* = 3. (**A**) Steroid-synthesis-related protein bands and analysis. (**B**) Levels of estradiol and progesterone secretion. (**C**,**D**) Cumulus-diffusion- and apoptosis-related protein bands and analysis. (**E**–**H**) After RAPA treatment, *n* = 3. (**E**) Steroid-synthesis-related protein bands and analysis. (**F**) Levels of estradiol and progesterone secretion. (**G**,**H**) Cumulus-diffusion- and apoptosis-related protein bands and analysis, *n* = 3. Values are mean ± SE, *: *p* < 0.05, **: *p* < 0.01, NS: no difference. Original Western blot images are provided in the Supplementary Materials.

To illustrate the effect of CIRBP on the function of YCCs by regulating mitophagy, we co-treated YCCs with PEX-CIRBP and 3MA and detected the expression of E2 and P4 synthesis-, cumulus-diffusion- and apoptosis-related proteins. We found that mitophagy inhibits significant hypocretin hormone synthesis and cumulus diffusion. CIRBP overexpression reversed the decreased expression of E2 and P4 biosynthesis- and cumulus-diffusion-related proteins caused by mitophagy inhibition (Figure 6A–D). Meanwhile, CIRBP overexpression significantly alleviated the mitophagy-inhibition-induced down-regulation of Bcl-2/BAX ratio. The ELISA results were consistent with the WB results. CIRBP overexpression significantly repaired 3MA-inhibited E2 and P4 secretion. In addition, we treated YCCs with RAPA and found that mitophagy activation showed similar results to those of CIRBP overexpression (Figure 6G,H). In summary, our data showed that CIRBP improve YCC function by activating mitophagy to promote cumulus diffusion, increasing steroid hormone synthesis and secretion, and inhibiting apoptosis.

### 3.5. CIRBP Activated YCC Mitophagy Through the AMPK/mTOR Signaling Pathway

**Figure 7:**
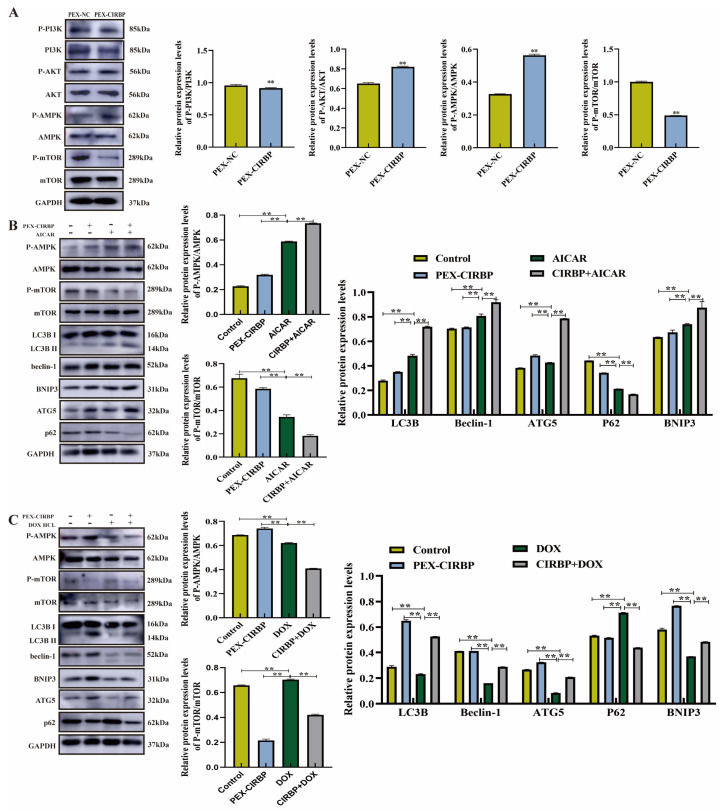
AMPK/mTOR signaling pathway mediates CIRBP regulation of mitophagy in YCCs. (**A**) AMPK/mTOR and PI3K/AKT pathways’ protein bands and analysis, *n* = 3. (**B**) Mitophagy-related protein bands and analysis after activation of AMPK/mTOR pathway, *n* = 3. (**C**) Mitophagy-related protein bands and analysis after inhibition of AMPK/mTOR pathway, *n* = 3. Values are mean ± SE, **: *p* < 0.01. Original Western blot images are provided in the Supplementary Materials.

AMPK, PI3K, AKT and mTOR are the main regulators of autophagy and mitophagy at the post-translational level. The AMPK/mTOR and PI3K/AKT signaling pathways are essential for the regulation of mitophagy and apoptosis [30]. To explore whether AMPK/mTOR- and PI3K/AKT-mediated mitophagy was activated by CIRBP overexpression, we further explored whether CIRBP overexpression activates AMPK/mTOR- and PI3K/AKT-mediated mitophagy. We found that the up-regulation of CIRBP induced p-AKT and p-AMPK and inhibited p-PI3K and p-mTOR, while the total protein levels of AKT, AKMP, PI3K and mTOR remained unchanged (Figure 7A). This suggests that CIRBP’s regulation of mitophagy may involve the AMPK/mTOR pathway rather than the PI3K/AKT signaling pathway.

To verify that CIRBP-activated mitophagy was mediated by the AMPK/mTOR pathway, we co-treated YCCs with the AMPK/mTOR pathway activators AICAR and PEX-CIRBP to detect mitophagy. The results showed that the AMPK/mTOR pathway was activated after AICAR treatment; the expression levels of mitophagy-related proteins LC3B, Beclin-1, ATG5, and BNIP3 increased; and the expression level of P62 was continuously inhibited (Figure 7B). CIRBP overexpression enhanced the role of AICAR in activating mitophagy. In addition, we added AMPK inhibitor DOX HCL to co-treat with PEX-CIRBP and found that DOX HCL inhibited the AMPK/mTOR pathway (Figure 7C), and pathway inhibition significantly down-regulated the expression levels of LC3B, Beclin-1 and BNIP3. Overexpression of CIRBP significantly alleviated the down-regulation of mitophagy-related proteins via AMPK/mTOR pathway inhibition, in contrast to the effects of AICAR. Our results showed that the AMPK/mTOR signaling pathway mediates CIRBP’s regulation of mitophagy in YCCs.

### 3.6. PI3K/AKT Signaling Pathway Mediates CIRBP’s Regulation of Apoptosis in YCCs

**Figure 8:**
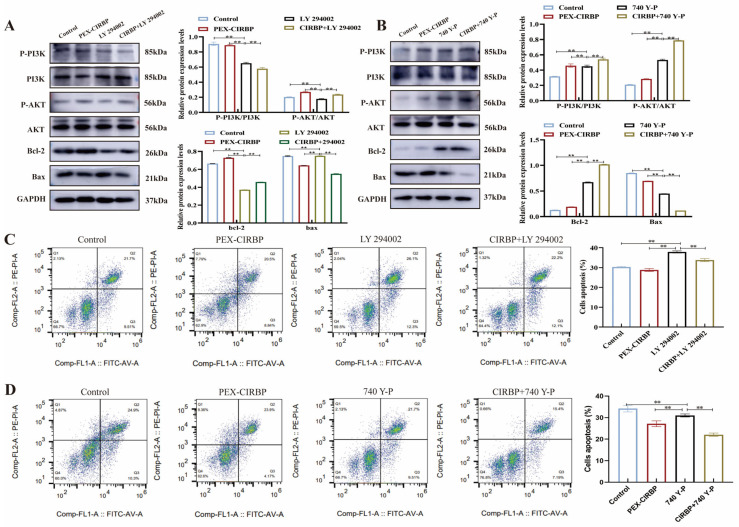
PI3K/AKT signaling pathway mediates CIRBP regulation of apoptosis in YCCs. (**A**) Associated protein bands and analysis after co-treatment of PEX-CIRBP with LY 294002, *n* = 3. (**B**) Associated protein bands and analysis after co-treatment of PEX-CIRBP with 740 Y-P. (**C**,**D**) Flow of apoptosis after co-treatment of PEX-CIRBP with LY 294002 and 740 Y-P assay, *n* = 3; Values are mean ± SE, **: *p* < 0.01,. Original Western blot images are provided in the Supplementary Materials.

Previous studies have shown that overexpression of CIRBP inhibits apoptosis and activates the PI3K/AKT pathway. We speculate that the anti-apoptotic effect of CIRBP may be related to its activation of the PI3K/AKT pathway. Therefore, we regulated the PI3K/AKT pathway on the basis of CIRBP overexpression and detected the level of apoptosis to verify the speculation above. We found that when the PI3K/AKT pathway was inhibited, BAX expression was up-regulated, Bcl-2 expression was down-regulated, and apoptosis was activated (Figure 8A,B). However, overexpression of CIRBP significantly alleviated the down-regulation of Bcl-2 by PI3K signal transduction pathway inhibition, compared to the blank control group, when the PI3K/AKT pathway was activated by 740-YP treatment; thus, CIRBP overexpression showed a synergistic effect with 740 Y-P treatment. Overexpression of CIRBP increased the induction of Bcl-2 via activation of the PI3K/AKT pathway and significantly inhibited Bax expression. In addition, we found that overexpression of CIRBP significantly alleviated the apoptosis induced by ly294002 and increased the anti-apoptotic effect of 740Y-P (Figure 8C,D). These data suggest that the regulation of CIRBP of the apoptosis of YCCs involves the PI3K/AKT signaling pathway.

## 4. Discussion

CIRBP is one of the RNA-binding proteins that can quickly respond to the up-regulation of low-temperature stress and participate in the stable cell state [31]; the optimal expression temperature range of CIRBP is about 32–34 °C. It is mainly located in the nucleus, and it participates in regulating gene transcription or binding with mRNA for post-transcriptional regulation [32]. However, under physiological or stress conditions, the expression site of CIRBP can migrate and even be released into the extracellular environment to regulate the transcription of target genes [32]. Research shows that under low-temperature conditions, transcription factor specificity protein 1 (Sp1) binds to the CIRBP promoter and activates its expression, while overexpression of Sp1 leads to the translocation of CIRBP from the cell nucleus to the cytoplasm [33]. This indicates that the function of CIRBP is determined by its expression location. As a functionally stable mRNA-binding protein, its expression location is related to its transcription method and the conditions regulating its expression level. It has been reported that a therapeutic low temperature (32 °C) can exert neuroprotective effects by activating CIRBP expression and inhibiting the production of mitochondrial apoptotic factors [34,35]. Expression of the CIRBP gene in bovine oocytes and cumulus cells is induced to be up-regulated by mild hypothermia [11]. Consistent with the findings above, we found that a small amount of CIRBP protein expression sites migrated and that CIRBP expression was induced to be up-regulated in cumulus cells after a mild low-temperature (32 °C) treatment. Autophagy is an important mechanism for maintaining cellular homeostasis; it is a complex process that can engulf and recycle cellular debris in lysosomes to adapt to the cellular environment. Mitophagy refers to the quality control mechanism that selectively remove damaged mitochondria through autophagosomes. Mild hypothermia has been shown to activate cellular mitophagy. Weng w et al. [36] showed that mild hypothermia treatment down-regulated lactonization of Tufm (Tu translation elongation factor, mitochondrial), a key regulator of mitophagy, in mouse neuronal cells, and activated mitophagy by facilitating its interaction with mitochondrial membrane proteins. Tan YL et al. [37] found that mild hypothermia treatment accelerated mitochondrial biogenesis in damaged hepatocytes by activating mitophagy to improve mitochondrial function. In our research, mild hypothermia activated mitophagy in YCCs. In contrast, studies have shown that prolonged hypothermia inhibits autophagy in mouse cardiac cells, but autophagy levels are repaired upon recovery from hypothermia [38]. This indicates that the regulation of mitophagy by hypothermia is dynamic and may be related to the degree of hypothermia exposure, hypothermia time, and cell type. In addition, our data show that hypothermia significantly increases the secretion levels of E2 and P4 in YCC. Cholesterol is the basis for steroid hormone biosynthesis. The inhibition of autophagy can damage the biosynthesis of steroid hormones by reducing the phagocytosis and transport of cholesterol by lipid droplets [39]. Ti_3_C_2_ nanosheets activate autophagy in mouse GCs to promote the ability of ovarian hormone secretion, while RAPA treatment of granulosa cells exhibited similar results [40]. Notably, scholars pointed out that the expression trends of CIRBP and LC3B showed a correlation in the cells treated with hypothermia followed by rewarming, and LC3B was regulated by CIRBP [41]. In summary, we propose hypothermia-induced mitophagy activation and increased E2 and P4 secretion in YCCs. It is hypothesized that this change may be CIRBP-mediated.

We verified the speculation above and found that up-regulation of CIRBP was accompanied by increased mitophagy activation and E2 and P4 synthesis and secretion in YCCs. Evidence suggests that as a protective factor for mitochondrial homeostasis, CIRBP eliminates endogenous ROS to promote mitochondrial metabolism [42]. CIRBP can induce ROS release and mtDNA fragmentation through TLR4 signaling, thereby activating macrophage mitophagy and inhibiting apoptosis [21]. CIRBP down-regulation can reduce mitophagy by inhibiting the PINK1/Parkin pathway [43]. Under stressful conditions, CIRBP activates ferritin autophagy by interacting with ELAVL1 to alleviate necroptosis [7]. In our study, BNIP3, LC3B and Beclin-1 were all up-regulated by CIRBP, highlighting the regulation of CIRBP on mitophagy in YCCs. This evidence also suggests that CIRBP has a regulatory role in apoptosis. Related studies found that down-regulation of CIRBP resulted in impaired ROS metabolism and activation of the PHD3/HIF-1α pathway to promote apoptosis [15]. CIRBP alleviates chemotherapy-induced cardiomyocyte apoptosis by inhibiting the OGF/OGFR axis [44]. We found that up-regulation of CIRBP inhibited YCC apoptosis. In addition, our results revealed that CIRBP overexpression increased the expression of cumulus-diffusion-related proteins HAS2, PTGS2, PTX3, and TNFAIP6, demonstrating for the first time that CIRBP is a regulator of cumulus diffusion. The cumulus diffusion process affects oocyte development and ovulation efficiency, which are important for oocyte maturation and ovulation. Notably, the synthesis and metabolism of estradiol in cumulus cells are regulated by autophagy activated by exosomes from yak follicular fluid [45]. The inhibition of apoptosis and activation of autophagy alleviate the decrease in the gene expression levels of sheep cumulus-diffusion-related factors caused by zearalenone (ZEN) [46]. This indicates that autophagy regulates steroid hormone synthesis, apoptosis, and cumulus diffusion. Therefore, we speculated that the effects of CIRBP on E2 and P4 biosynthesis, cumulus diffusion, and apoptosis in YCCs may be mediated by mitophagy.

In this study, we investigated whether CIRBP can regulate the function of YCCs through mitosis. We found that overexpression of CIRBP could reverse the inhibitory effect of mitophagy inhibitor on YCC mitophagy. CIRBP can alleviate the decrease in E2 and P4 secretion and the decrease in cumulus-diffusion-related protein expression caused by mitophagy inhibition. In addition, the inhibition of mitophagy significantly increased the expression level of Bax, whereas the overexpression of CIRBP alleviated the induction of apoptosis by inhibiting autophagy. Simultaneously, the changes in the function of YCCs after mitophagy activation showed the same results as when CIRBP was overexpressed. Bovine-follicular-fluid-derived exosomes promote steroid hormone secretion and inhibit apoptosis by inducing mitophagy in GCs [47]. Benzopyrene (BaP) can inhibit CL cell mitophagy through the ANT1-PINK1-Parkin pathway, thereby inhibiting P4 synthesis [48]. Consistent with these findings, we found that mitophagy activation promotes the synthesis and secretion of steroid hormones. In contrast, Ma Q et al. [49] found that dehydroepiandrosterone (DHEA) improved the function of rat ovarian granulosa cells by inhibiting mitophagy and AMPK-SIRT1 signaling. Cd exposure inhibits P4 synthesis in placental trophoblasts by activating PERK-regulated mitophagy [50]. This indicates that the regulation of mitophagy on steroid hormone biosynthesis is dynamic, which may be related to the feedback regulation between the two [51]. We have previously reported that CIRBP inhibits apoptosis by activating mitophagy in other cells, and CIRBP also plays this role in yak YCCs. In addition, we found that CIRBP promoted cumulus diffusion by activating YCC mitophagy. A recent study showed that HIF-1α regulates the function of YCCs and participates in oocyte development [26]. HIF-1α has been proven to be one of the target genes of CIRBP [52]. However, whether the effect of CIRBP on cumulus diffusion is mediated by HIF-1α and its specific mechanism need to be further explored. In summary, our data show that CIRBP affected the biosynthesis and secretion of E2 and P4, cumulus diffusion, and apoptosis of YCCs by regulating mitophagy, thereby improving YCC function; these results emphasize the importance of exploring the regulation of cellular functions by CIRBP.

AMPK/mTOR and PI3K/Akt pathways are two of the main pathways regulating mitophagy and apoptosis [53,54]. CIRBP promotes wound healing by activating AMPK [55] and regulates apoptosis and proliferation through the PI3K/AKT signaling pathway [56], suggesting that CIRBP can regulate cell function through the AMPK/mTOR and PI3K/AKT pathways. Our study found that CIRBP activated the AMPK/mTOR and PI3K/AKT signaling pathways. Further studies found that overexpression of CIRBP significantly alleviated the decrease in mitophagy-related protein expression caused by AMPK/mTOR pathway inhibition, while overexpression of CIRBP further enhanced the activation of mitophagy by AMPK/mTOR pathway activation. This indicates that the regulation of CIRBP of mitophagy of YCCs is mediated by AMPK/mTOR signaling pathway. BNIP3 directly binds to LC3B to initiate mitophagy [57]. Moreover, the overexpression of BNIP3 significantly induces the up-regulation of AMPK; however, there is evidence that the expression level of BNIP3 does not change significantly when AMPK is inhibited or activated [58]. In our study, CIRBP overexpression significantly up-regulated BNIP3 protein level, and activation of the AMPK pathway up-regulated BNIP3 expression. BNIP3 is a target gene directly regulated by HIF-1α [59], and HIF-1α is a target gene of CIRBP. Therefore, we speculate that the HIF-1α/BNIP3 signaling pathway may mediate CIRBP regulation of mitophagy. Berberine inhibits cardiomyocyte apoptosis induced by regulating HIF-1α/BNIP3-pathway-mediated mitophagy [60]. This suggests that the inhibitory effect of CIRBP overexpression on the apoptosis of YCCs may be related to an increase in BNIP3 expression. In addition, our results indicate that the regulation of CIRBP on YCC cell apoptosis is mediated by the AKT signaling pathway. Low-temperature exposure promotes platelet activation by up-regulating CIRBP to activate the P-AKT signaling pathway, thereby increasing the risk of ischemic stroke [61]. Furthermore, CIRBP alleviates hepatocyte apoptosis induced by low-temperature exposure by activating the AKT signaling pathway [62]. In this study, we found that the AMPK/mTOR pathway mediated the regulation of mitochondrial autophagy in YCCs by CIRBP, and the AKT signaling pathway mediated the regulation of apoptosis in YCCs by CIRBP; combined with the previous results of this study, we demonstrated that CIRBP can regulate mitophagy through the AMPK/mTOR pathway to affect YCC function and alleviate apoptosis. These findings provide a reference for further exploration of CIRBP regulation of programmed cell death and the role of CIRBP in reproductive medicine.

## 5. Conclusions

In summary, this study found that CIRBP improved cumulus cell function by induction of AMPK/mTOR-pathway-mediated mitophagy and inhibiting AKT-pathway-mediated YCC apoptosis.

## Figures and Tables

**Table 1:** Specific primer sequences.

Primer	Primer Sequence (5′-3′)	Bp	Annealing Temperature
CIRBP	F: TGGATACCGTGGTGGCTCT R: GGCTGCTGTAGTAGTCTCTGG	161	60 °C
LC3B	F: TGTTAGGTCAGGCAGTCA R: GTAGTAGGAAGCACTCGTTA	150	56 °C
P62	F: ATCAGCCTCTGGTCCATC R: TTCTCTTGCCTCCGTGTT	128	56 °C
ATG5	F: ATCAATCGGAAACTCATG R: AGATGTTCACTCAGCCAC	272	56 °C
Beclin-1	F: AACCTCAGCCGAAGACTA R: TCAGCCTCTCCTCCTCTA	253	56 °C
STAR	F: GGACCTTGATCTCCTTGAC R: CCACACTCTATGAGGAGATG	178	56 °C
CYP1A1	F: GTCCCCTTCACCATCCCA R: CCAAGCCGAAAATAATCACC	194	56 °C
CYP1B1	F: GCTTCCGTCTTGGGCTAC R: GGTCAAAGTCCTCTGGGTTC	196	56 °C
CYP11A1	F: TTTGCCTTTGAGTCCATC R: CCTAAATTCTGTTTTCCGTC	273	56 °C
CYP17A1	F: GCCCAAGACCAAGCACTC R: GGAACCCAAACGAAAGGA	160	56 °C
CYP19A1	F: GAAGCACAGTCACTACATATC R: ATGGAATCAGCACAGATGG	183	56 °C
β-actin	F: CGTCCGTGACATCAAGGAGAAGC R: GGAACCGCTCATTGCCGATGG	141	56 °C
HAS2	F: ACAGGCATCTAACGAACCGAG R: AGTAGGACTTGCTCCAGCGG	135	56 °C
PTX3	F: GCTATCGGTCCATAATGCTTG R: CCACCGAGTCACCATTTACC	150	56 °C
COX2	F: CGTTTTCTCGTGAAGCCCTAT R: CAGTACTCGGGAGAGCATATAGG	230	56 °C
TSG6	F: AGCAGTTAGAGGCAGCCAGAAA R: AACACACCACCACACTCCTTTG	211	56 °C

## Data Availability

All the data obtained in this study can be obtained from the corresponding author under reasonable requirement.

## Supplementary Materials

The following supporting information can be downloaded at: https://www.mdpi.com/article/10.3390/biom15060759/s1. Original Western blot images are provided in the Supplementary Materials.

## List of Abbreviations

**PCD**	Programmed cell death
**GCs**	Granulosa cells
**ANOVA**	Analysis of variance
**YCCs**	Yak cumulus cells
**Sp1**	Specific protein 1
**MAPK**	Mitogen-activated protein kinase
**CYP**	Cytochrome P450 enzyme
**CIRBP**	Cold-induced RNA-binding protein
**HIF-1α**	Hypoxia-inducible factor 1α
**AMPK**	AMP-activated protein kinase
**TLR4**	Toll-Like Receptor 4
**BNIP3**	BCL2 interacting protein 3
**ROS**	Reactive oxygen species
**3-MA**	3-Methyladenine
**RAPA**	Rapamycin
**AICAR**	Acadesine
**DO** **X**	Doxorubicin
