# Supplementary material for: CIRBP Enhances the Function of Yak Cumulus Cells by Activating AMPK/mTOR-Mediated Mitophagy
Source: Biomolecules. 2025 May 24;15(6):759. doi: 10.3390/biom15060759 (PMC12190196; doi:10.3390/biom15060759)
Supplement: Supplementary file 1 [file biomolecules-15-00759-s001.zip › FIG 7.pdf]

FIG 7

FIG7 A

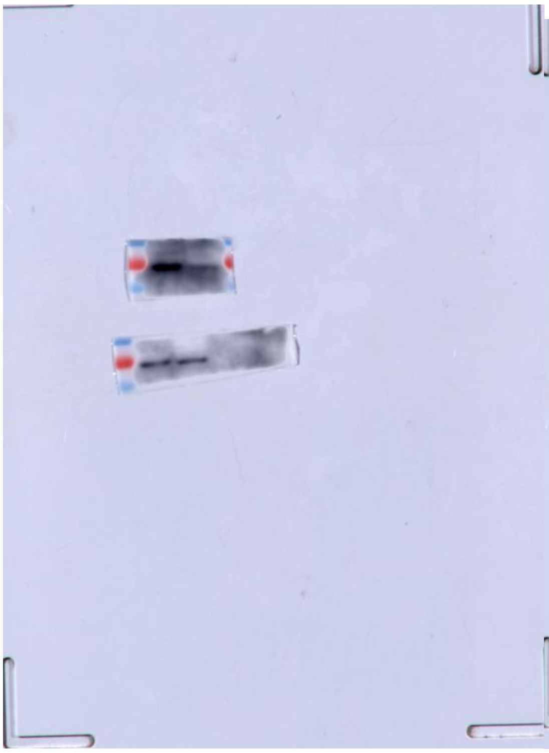

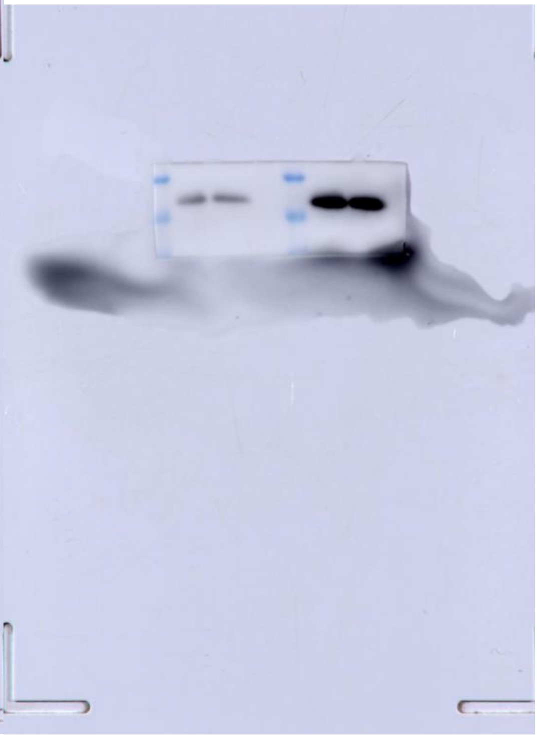

AMPK **GAPDH**

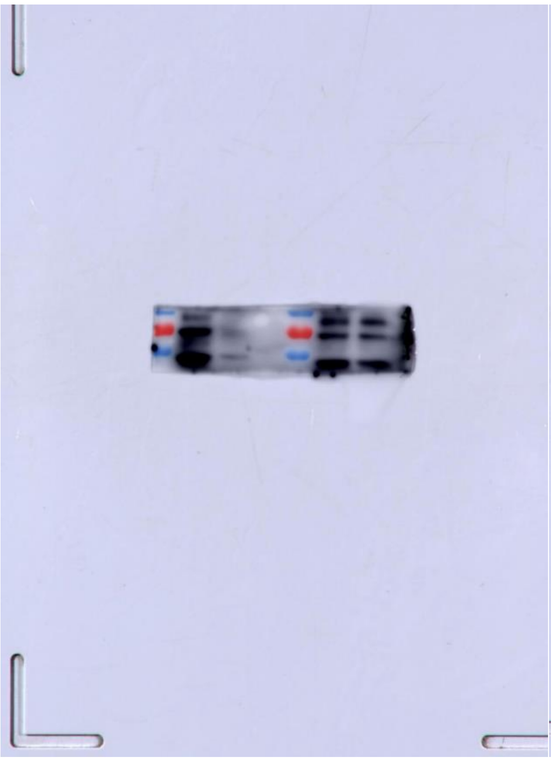

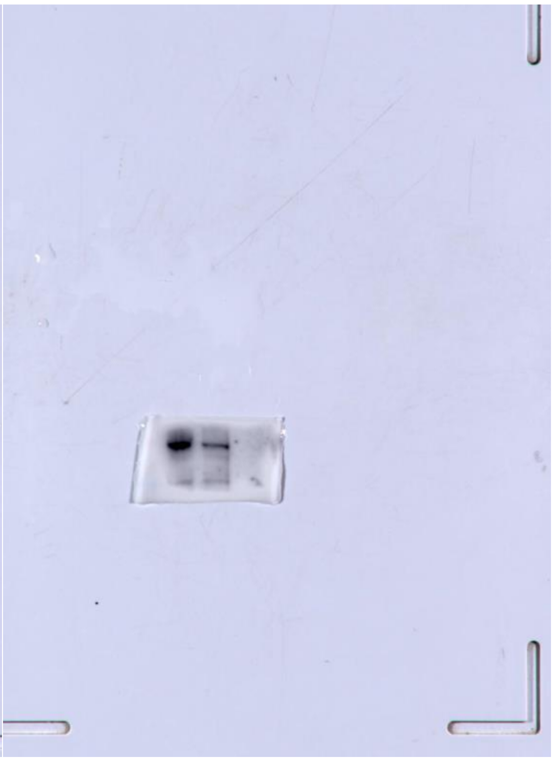

P-PI3K **P-mTOR**

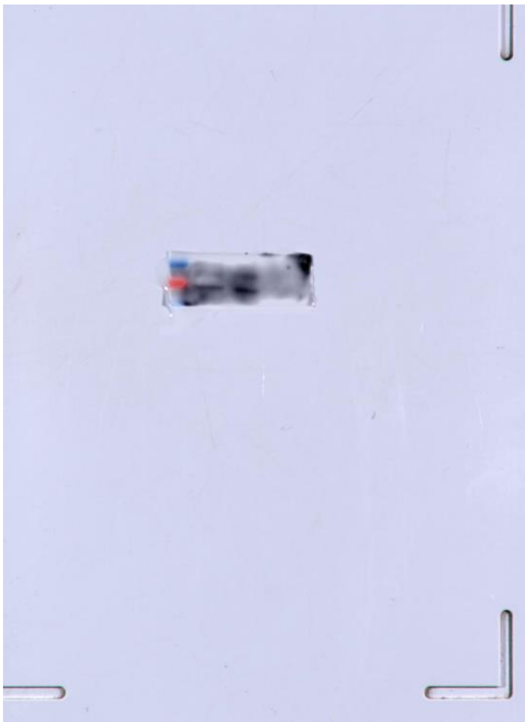

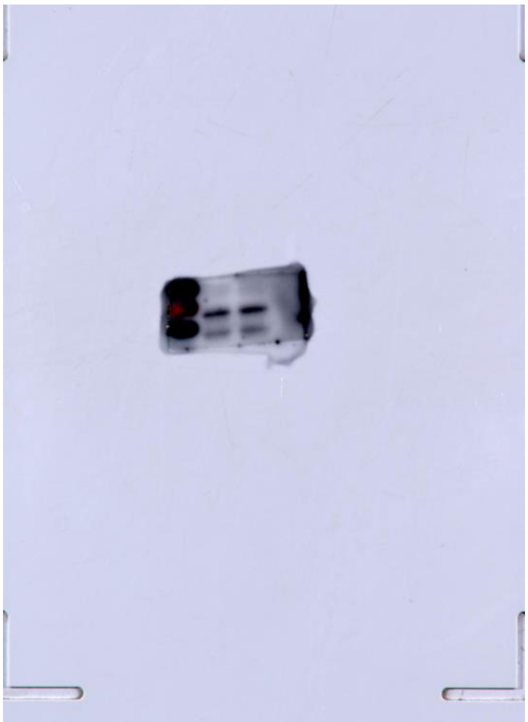

P-AMPK **P-AKT**

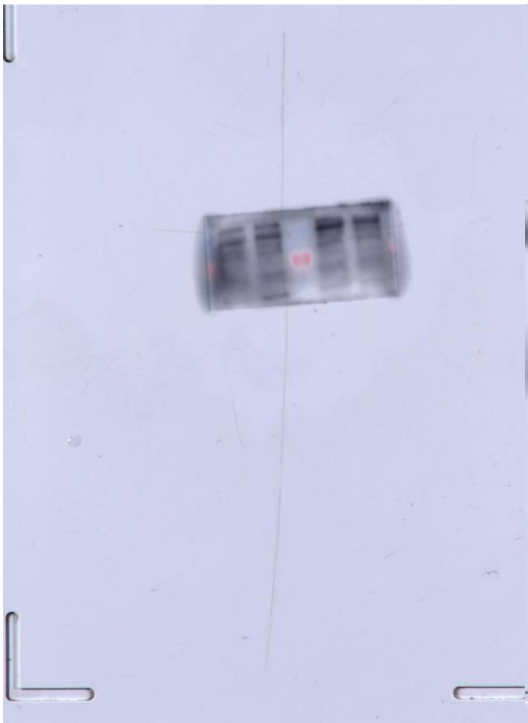

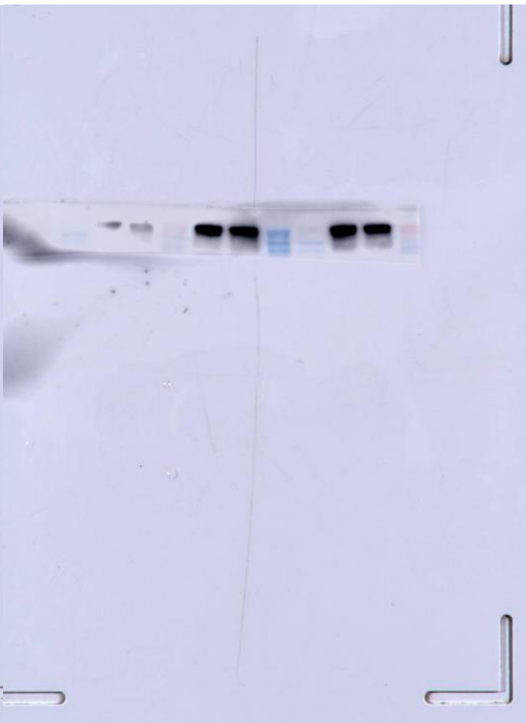

P-PI3K **mTOR**

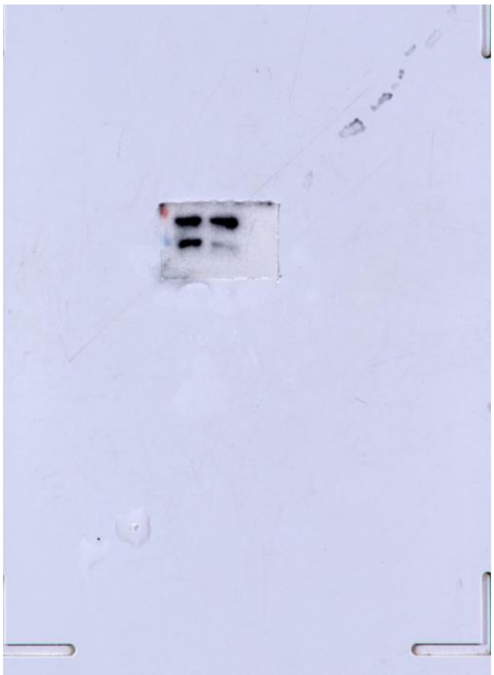

AKT

FIG7 B

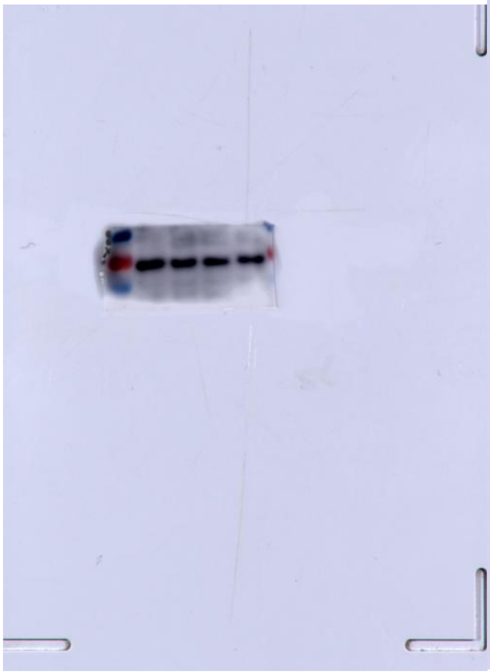

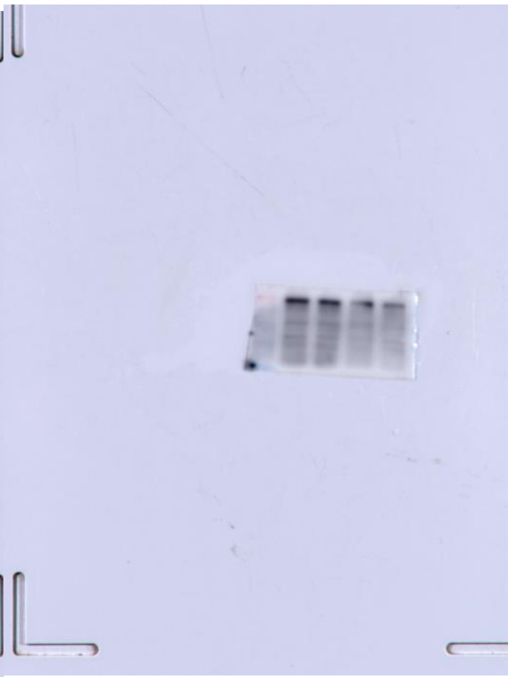

AMPK

P-mTOR

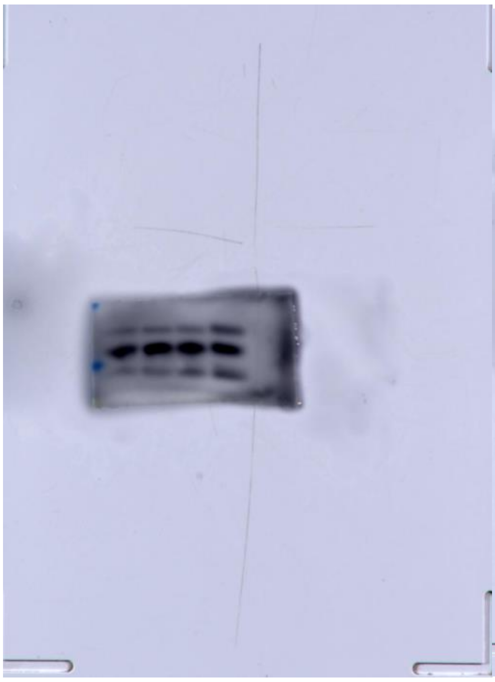

LC3B

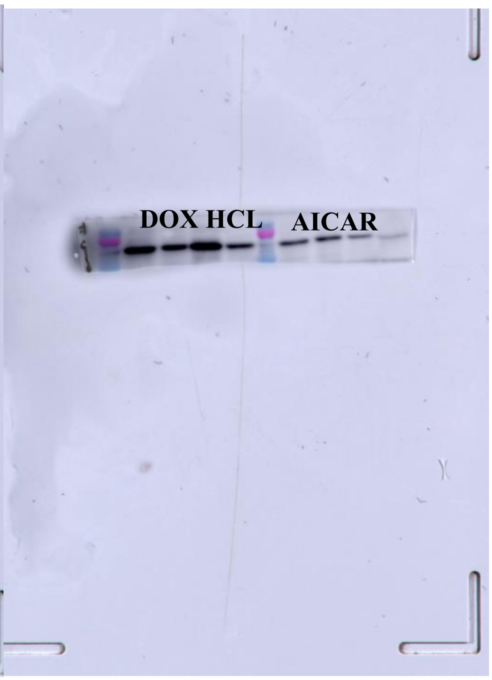

P62

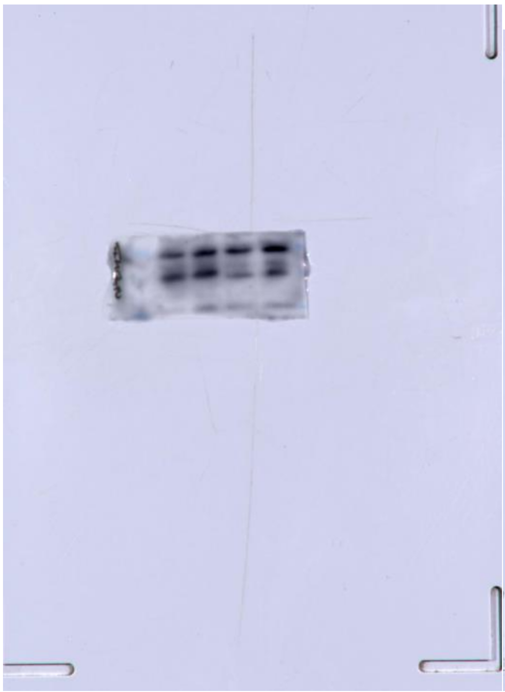

ATG5

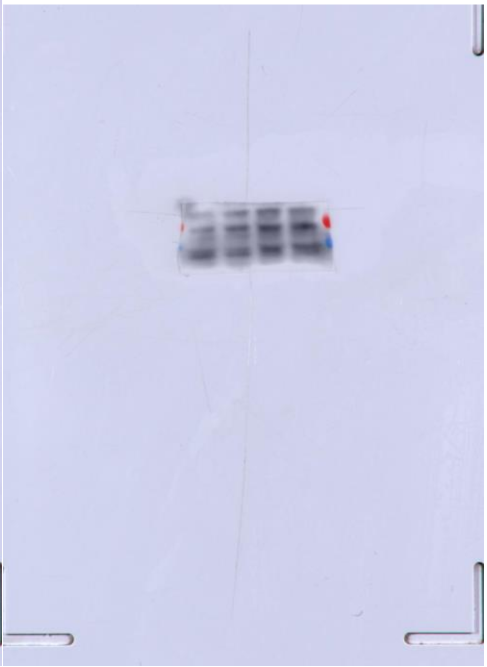

P-AMPK

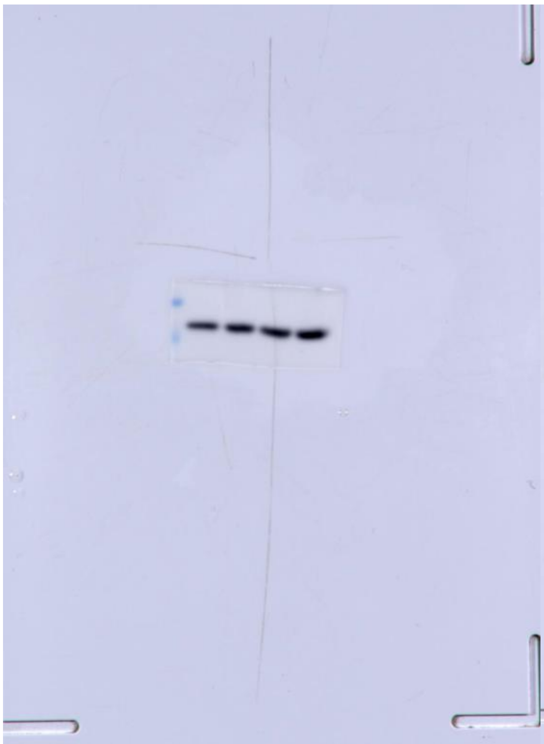

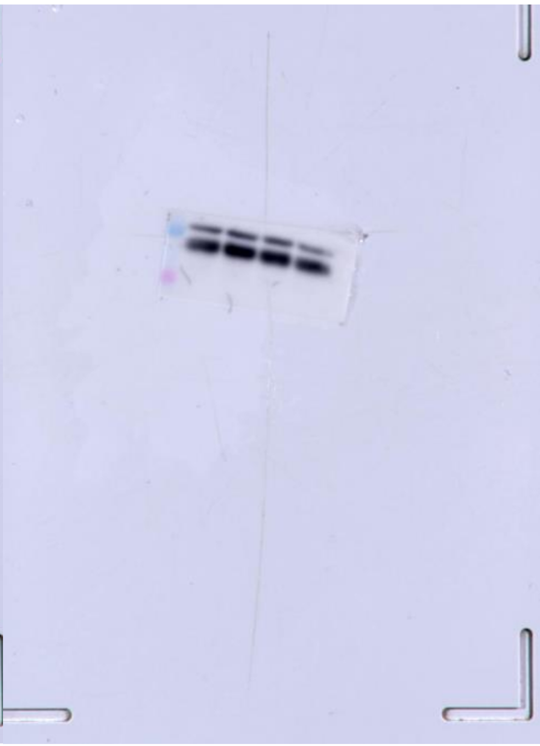

BNIP3 **GAPDH**

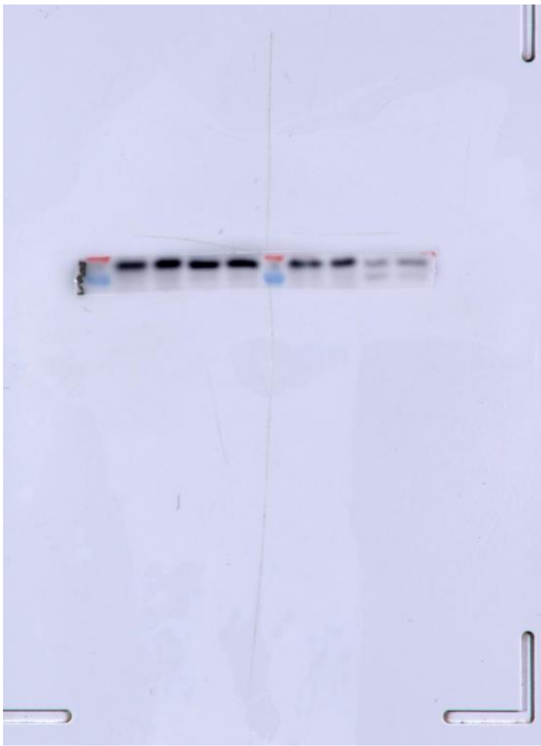

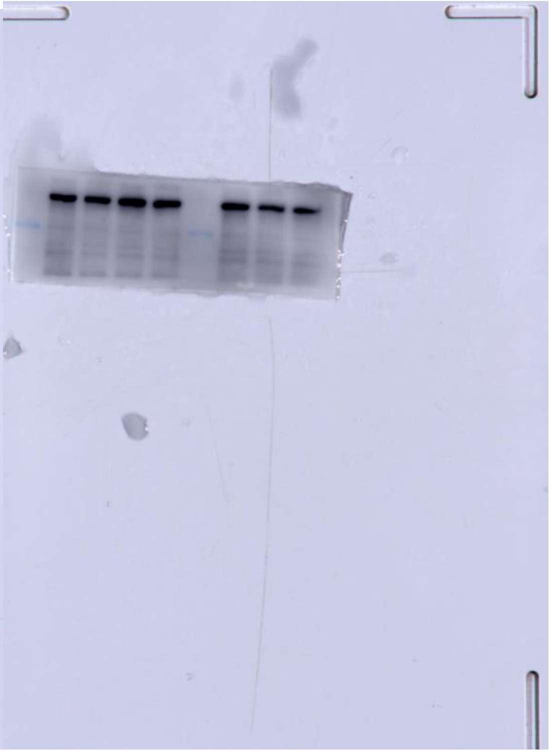

Beclin-1 **mTOR**

FIG7 C

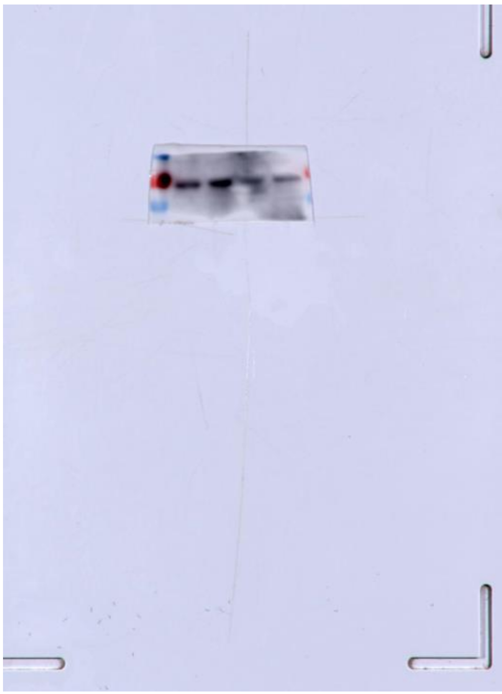

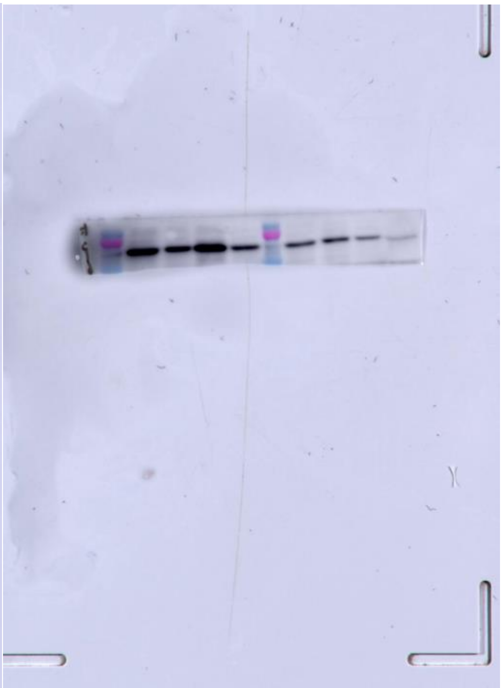

P-AMPK **P62**

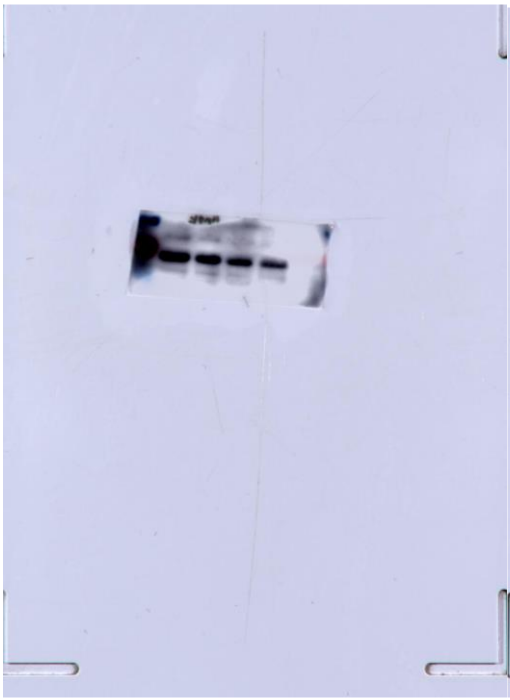

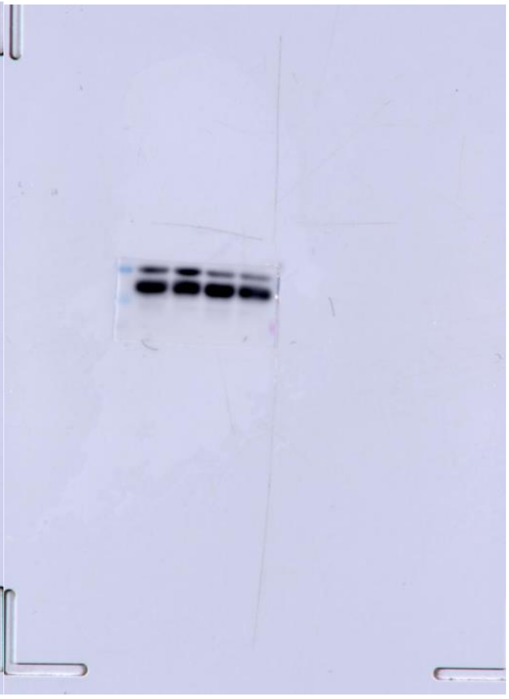

AMPK **GAPDH**

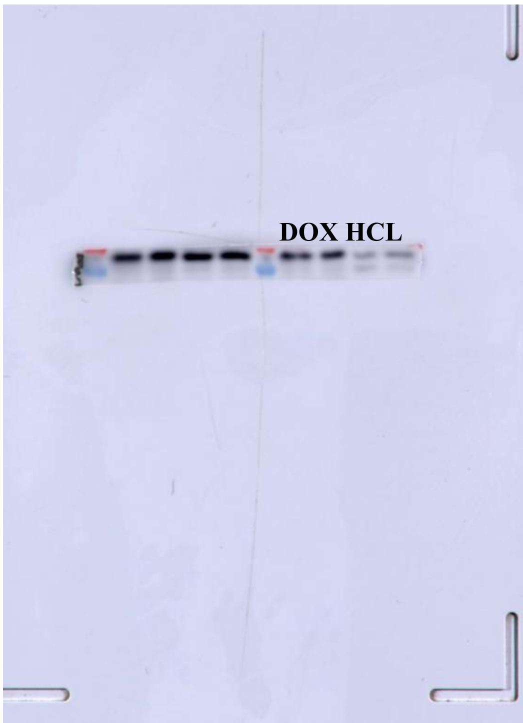

Beclin-1

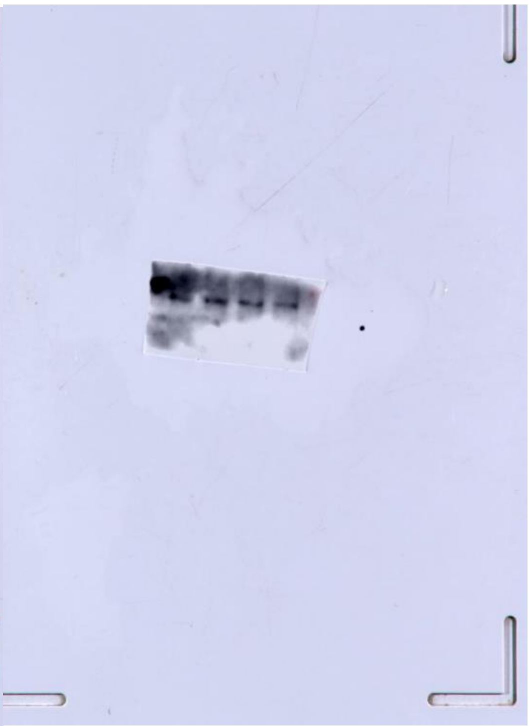

mTOR

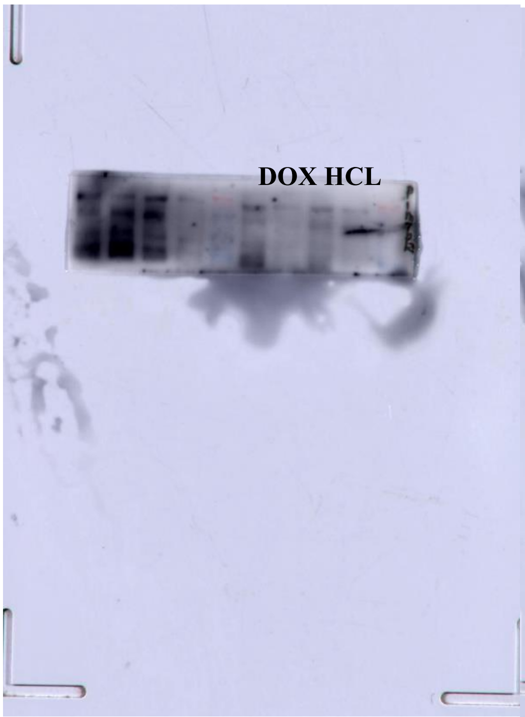

P-mTOR

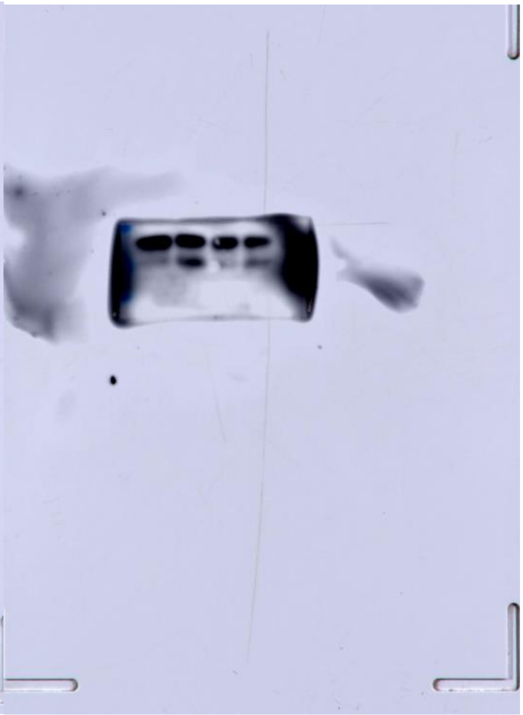

LC3B

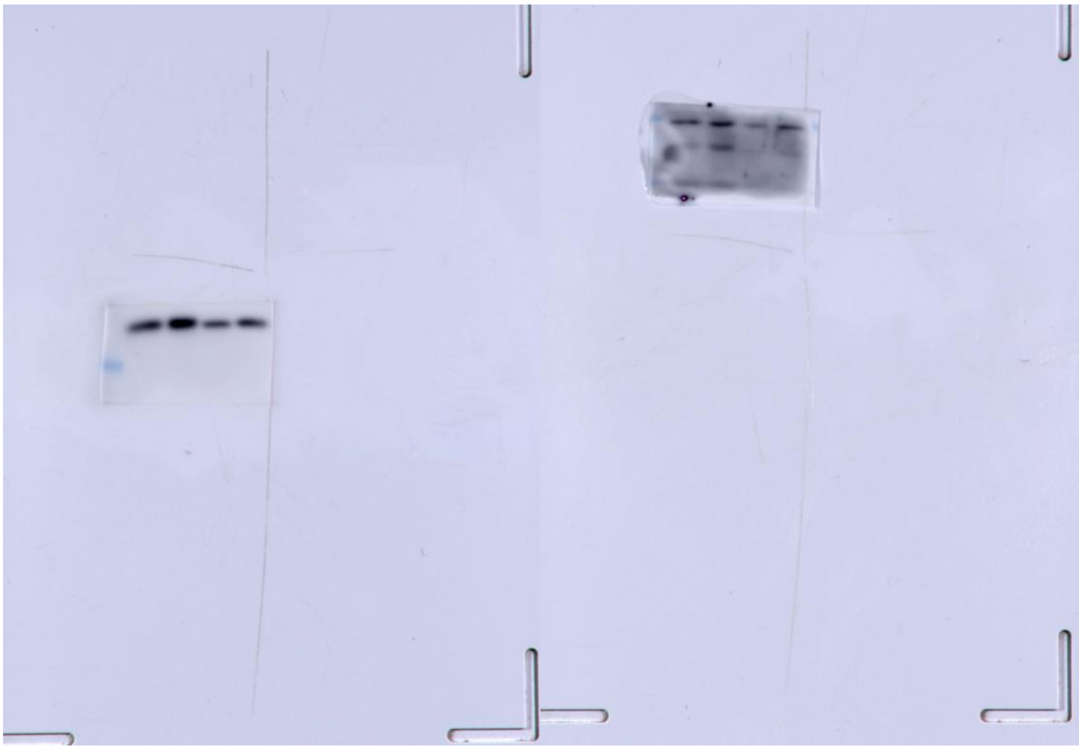

BNIP3 **ATG5**
