# Supplementary material for: CIRBP Enhances the Function of Yak Cumulus Cells by Activating AMPK/mTOR-Mediated Mitophagy
Source: Biomolecules. 2025 May 24;15(6):759. doi: 10.3390/biom15060759 (PMC12190196; doi:10.3390/biom15060759)
Supplement: Supplementary file 1 [file biomolecules-15-00759-s001.zip › FIG6.pdf]

FIG6

FIG6A

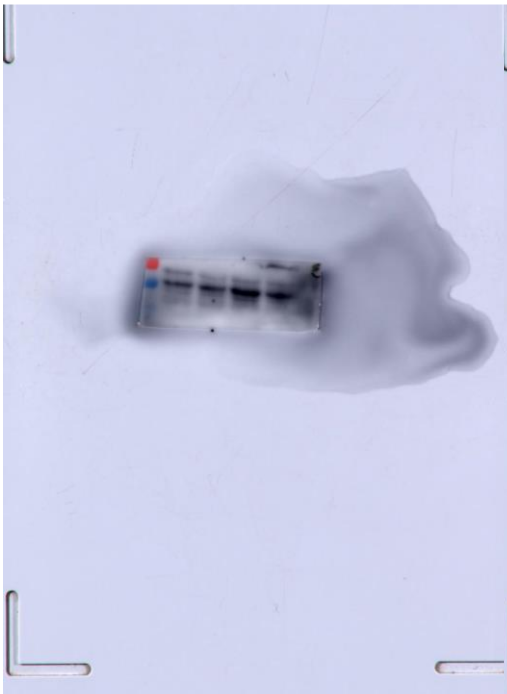

CYP19A1

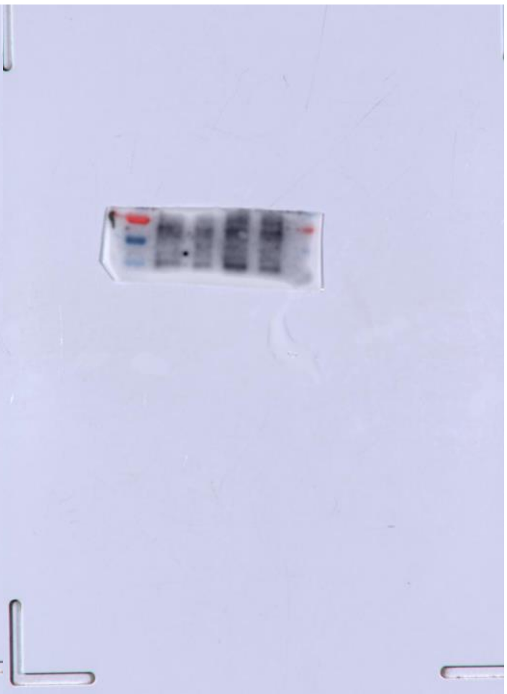

STAR

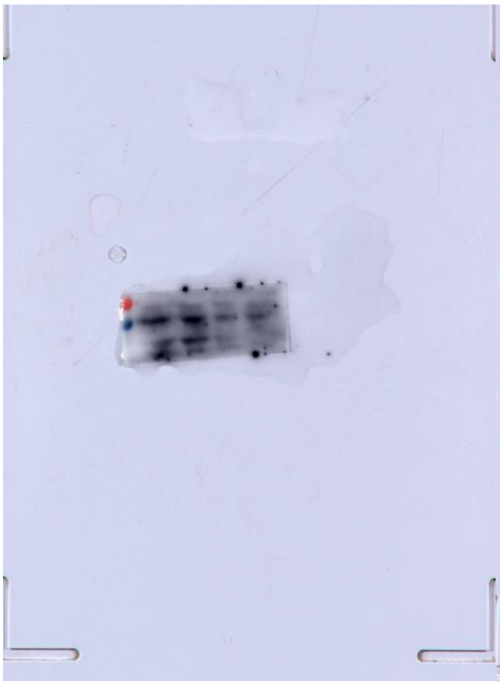

CYP17A1

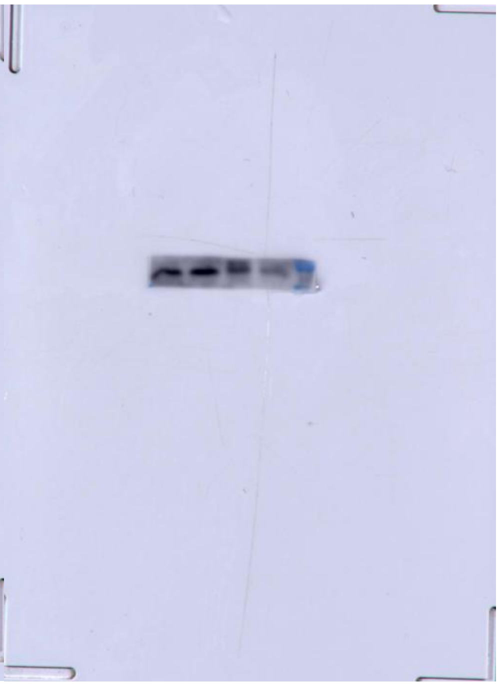

CYP1A1

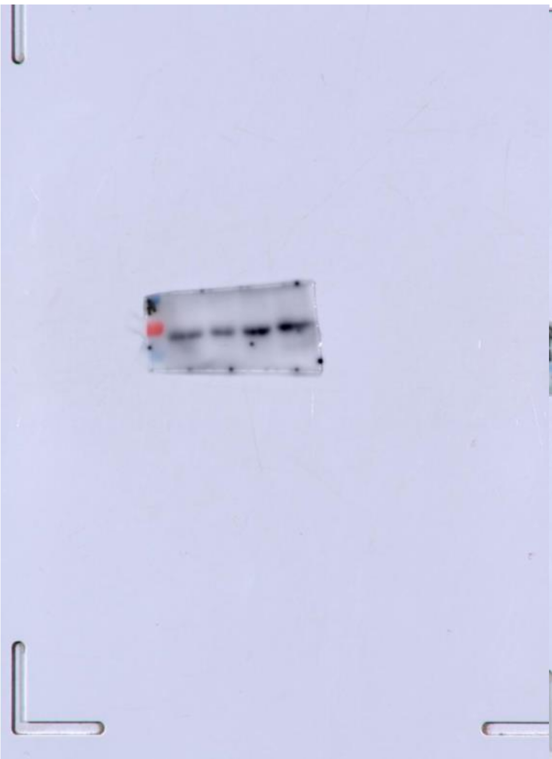

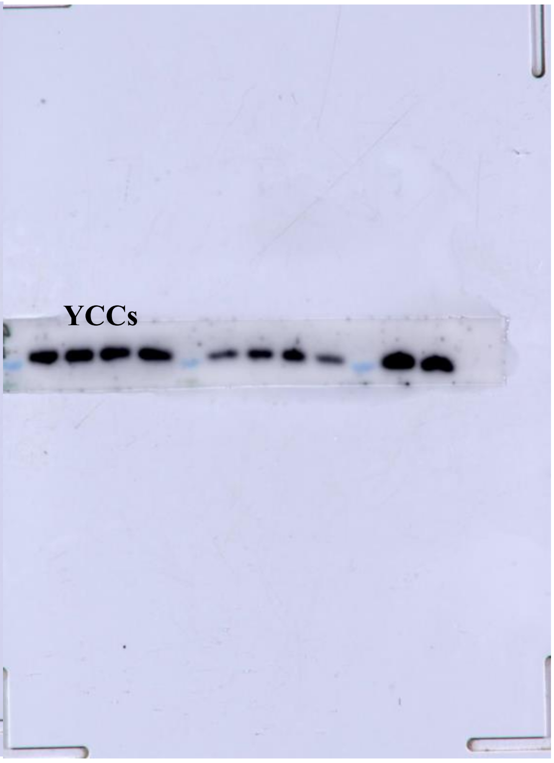

CYP1B1 **β-actin**

FIG6C

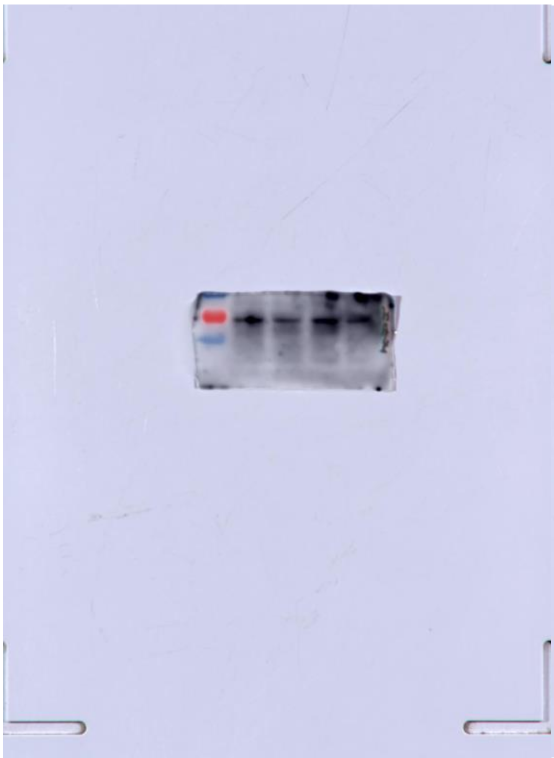

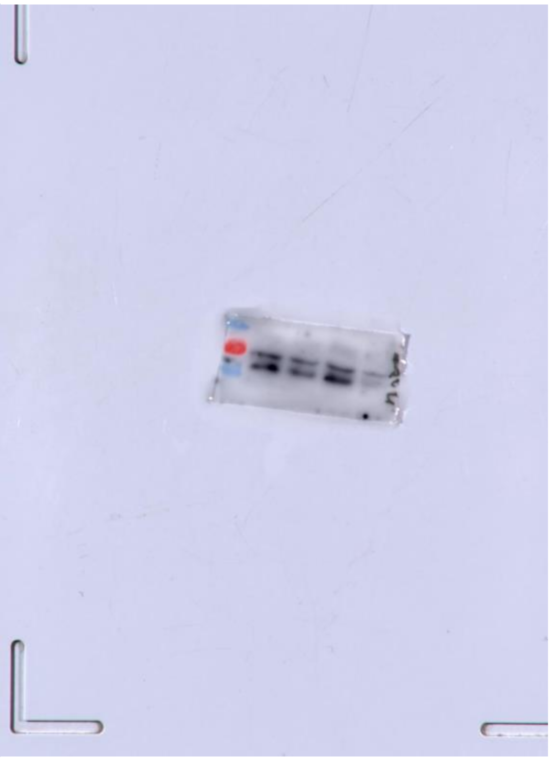

HAS2 **COX2**

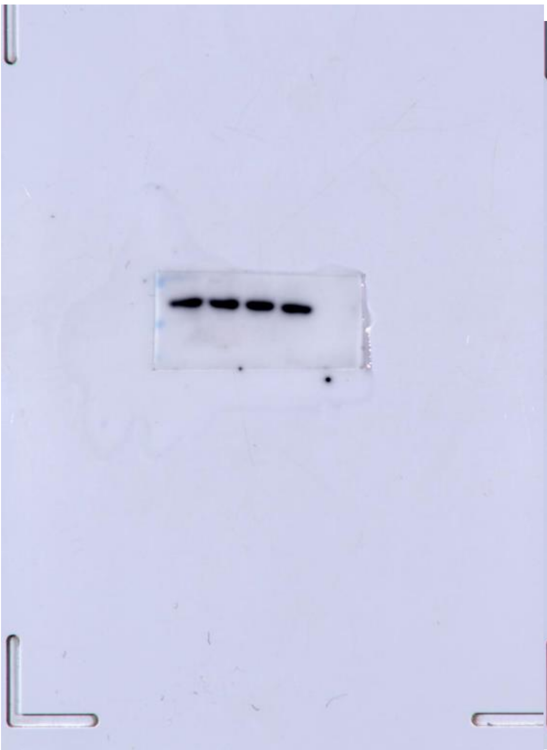

GAPDH

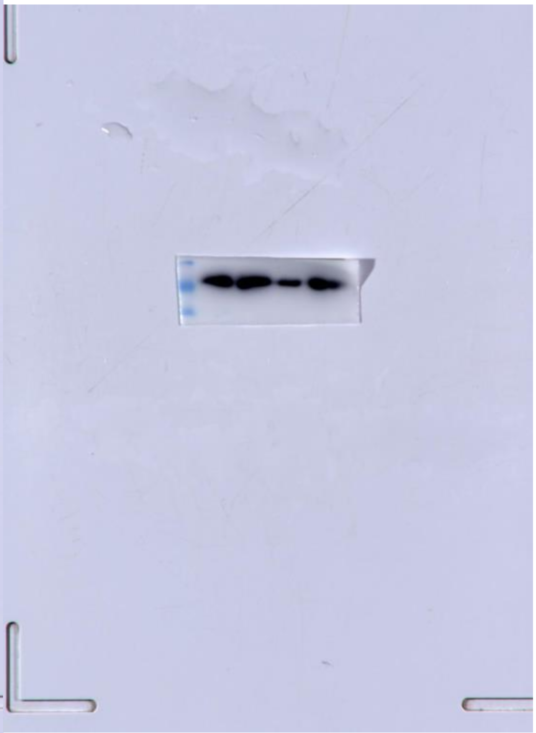

BAX

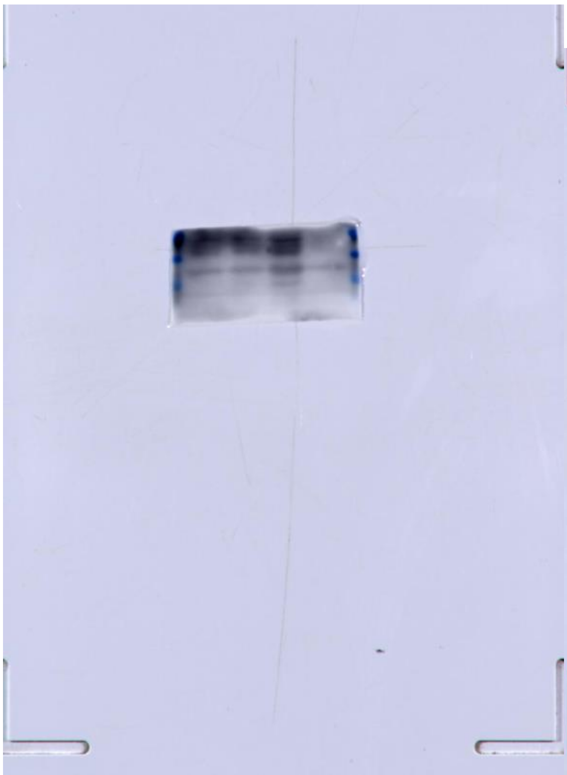

PTX3

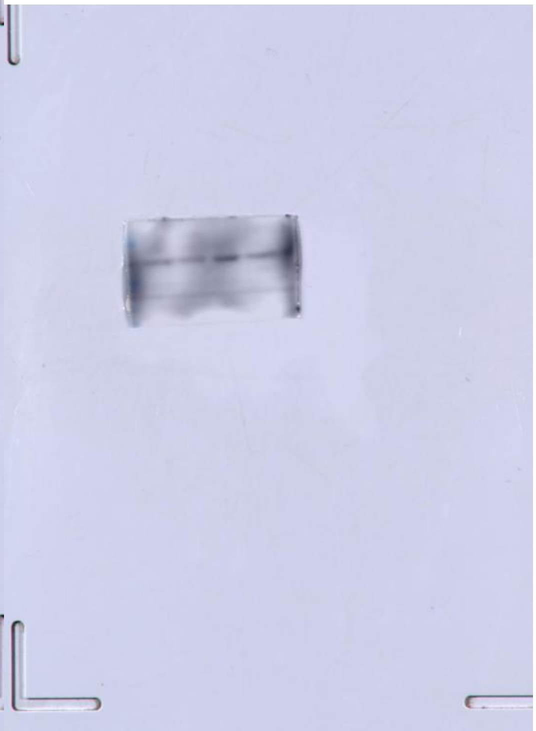

TSG6

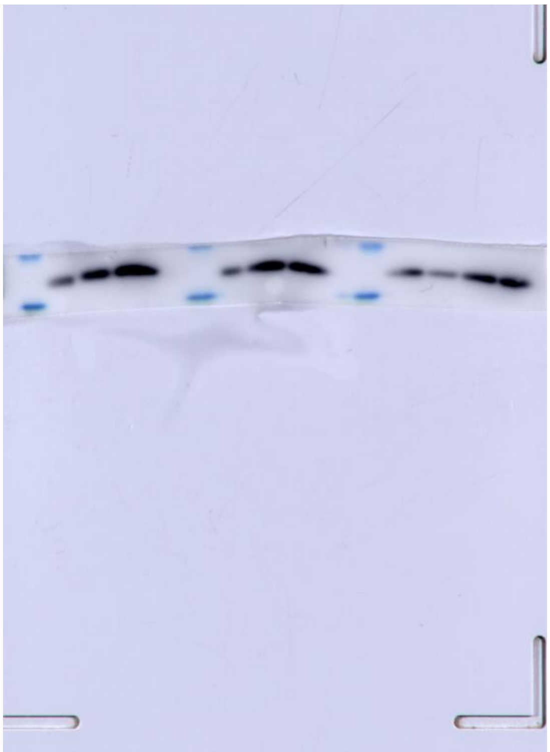

BCL2

FIG 6E

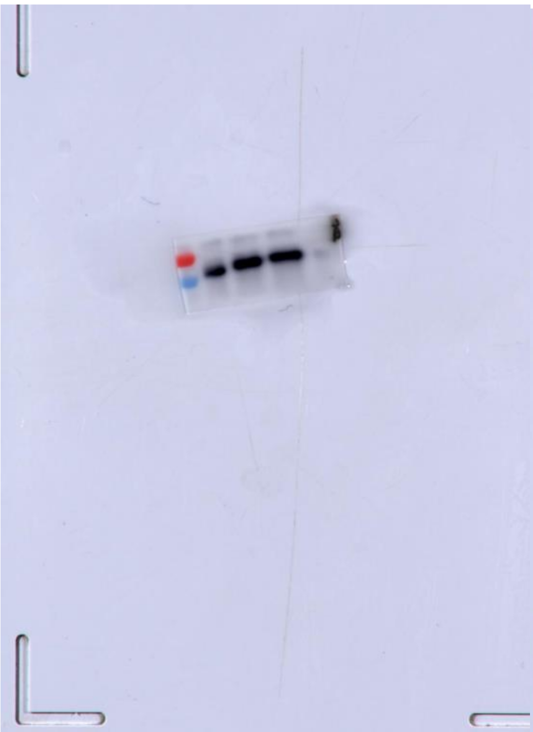

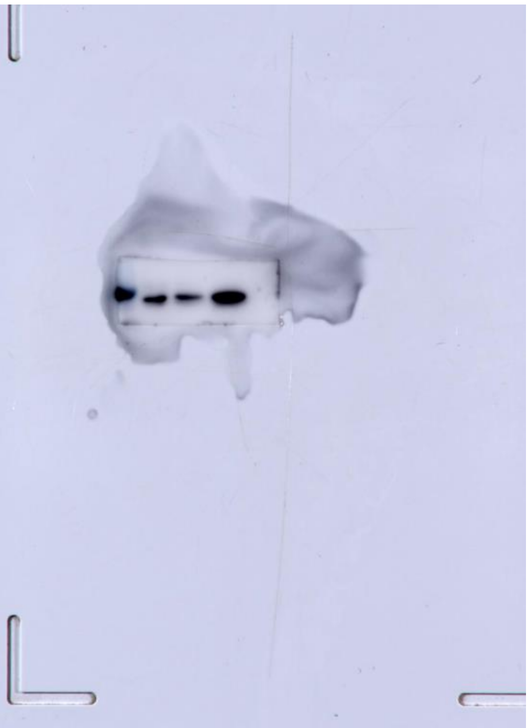

CYP19A1

CYP17A1

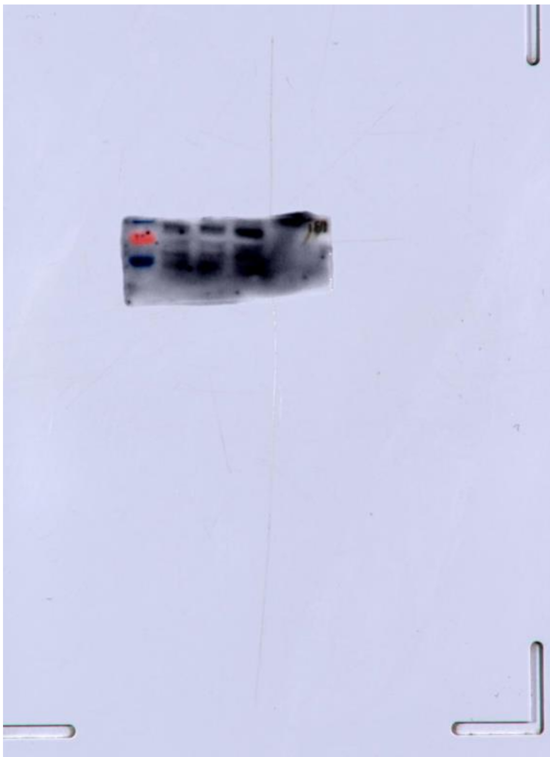

CYP1B1

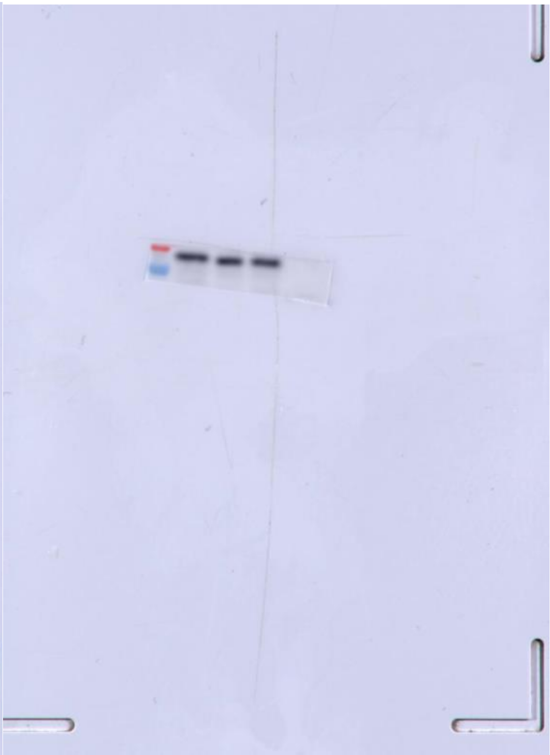

CYP1A1

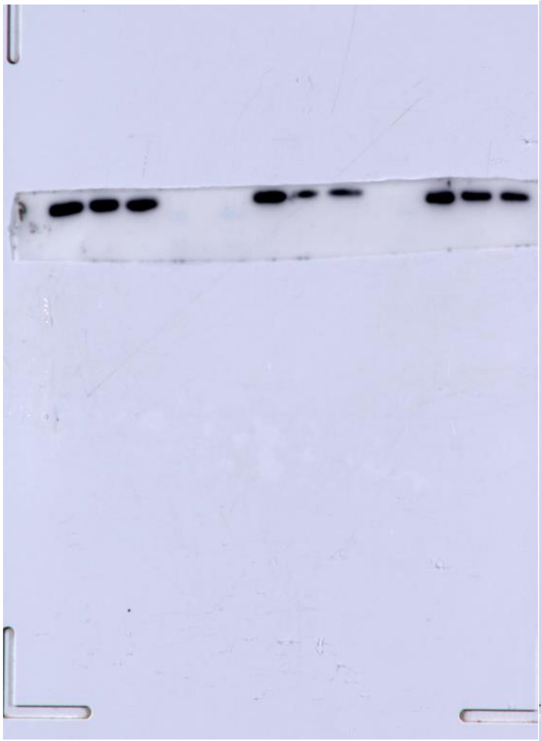

GAPDH

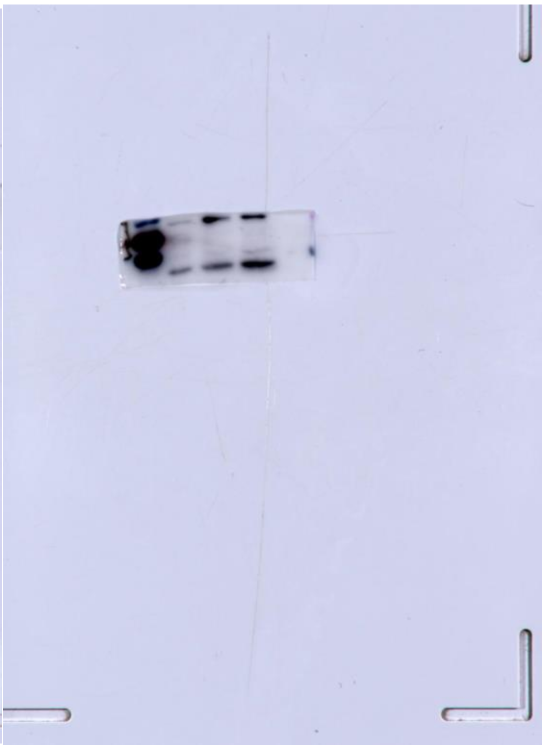

STAR

FIG6 G

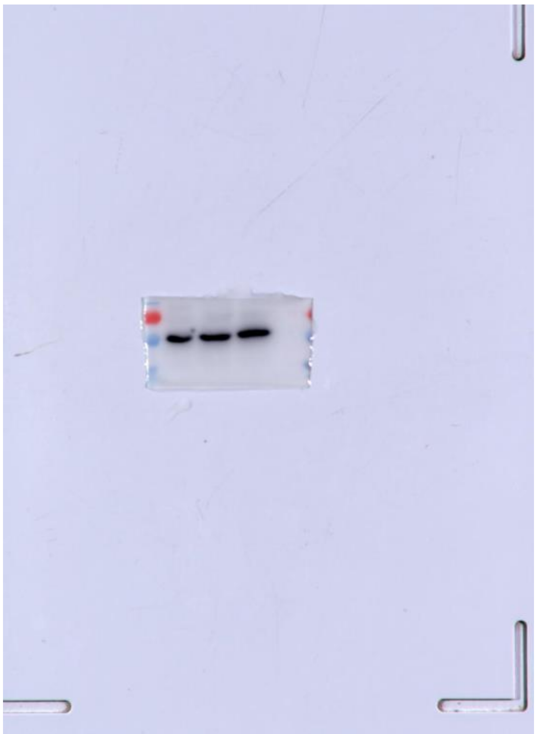

HAS2

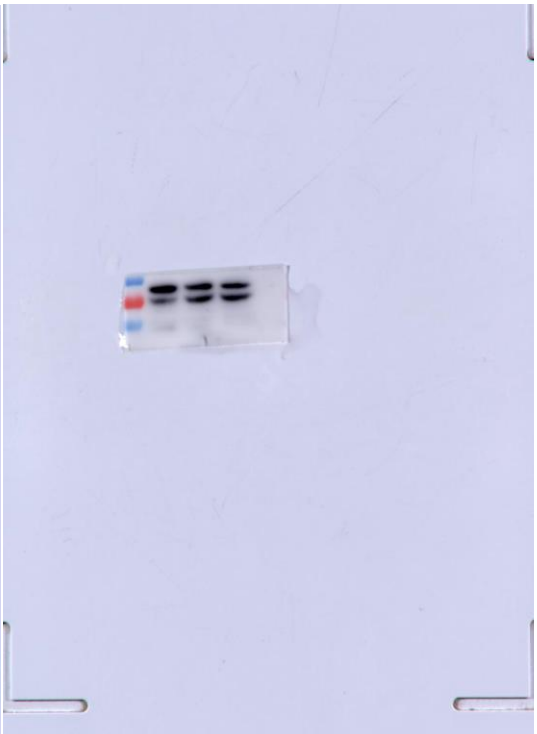

COX2

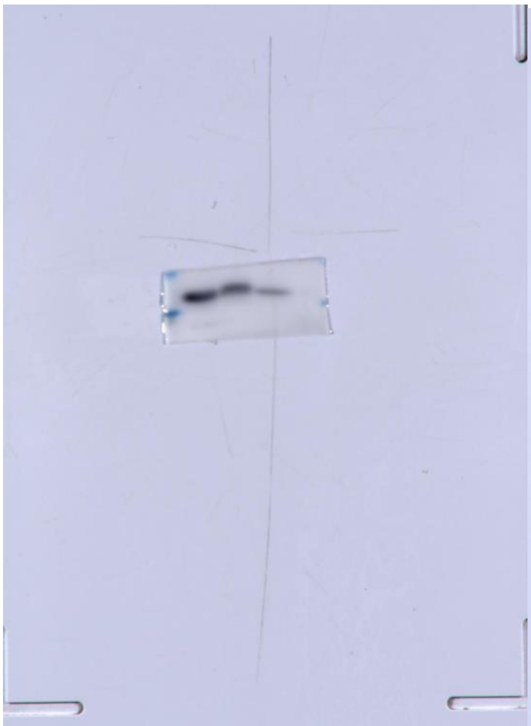

BAX

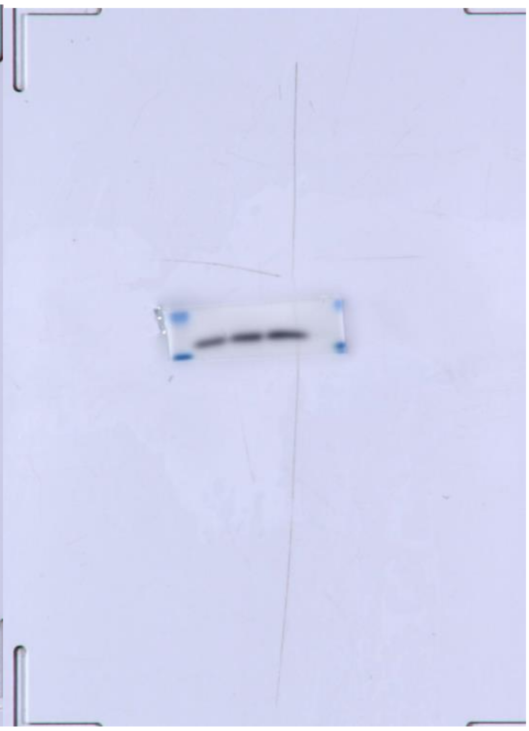

BCL-2

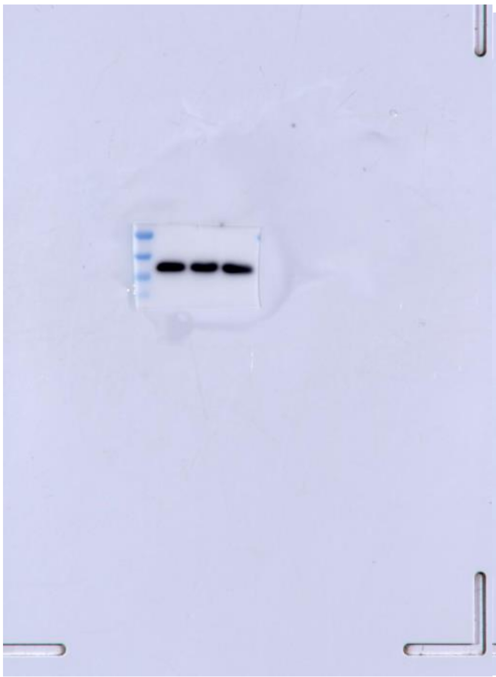

GAPDH

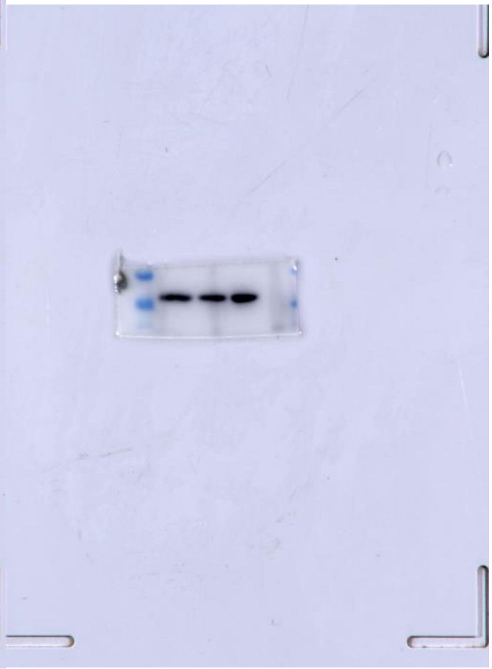

TSG6

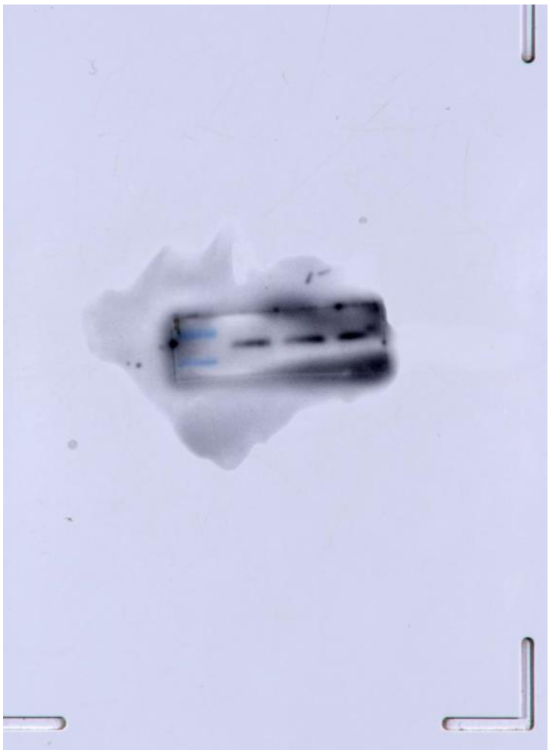

PTX3
