# Supplementary figures and images for: CIRBP Enhances the Function of Yak Cumulus Cells by Activating AMPK/mTOR-Mediated Mitophagy
Source: Biomolecules. 2025 May 24;15(6):759. doi: 10.3390/biom15060759 (PMC12190196; doi:10.3390/biom15060759)

FIG 1 C

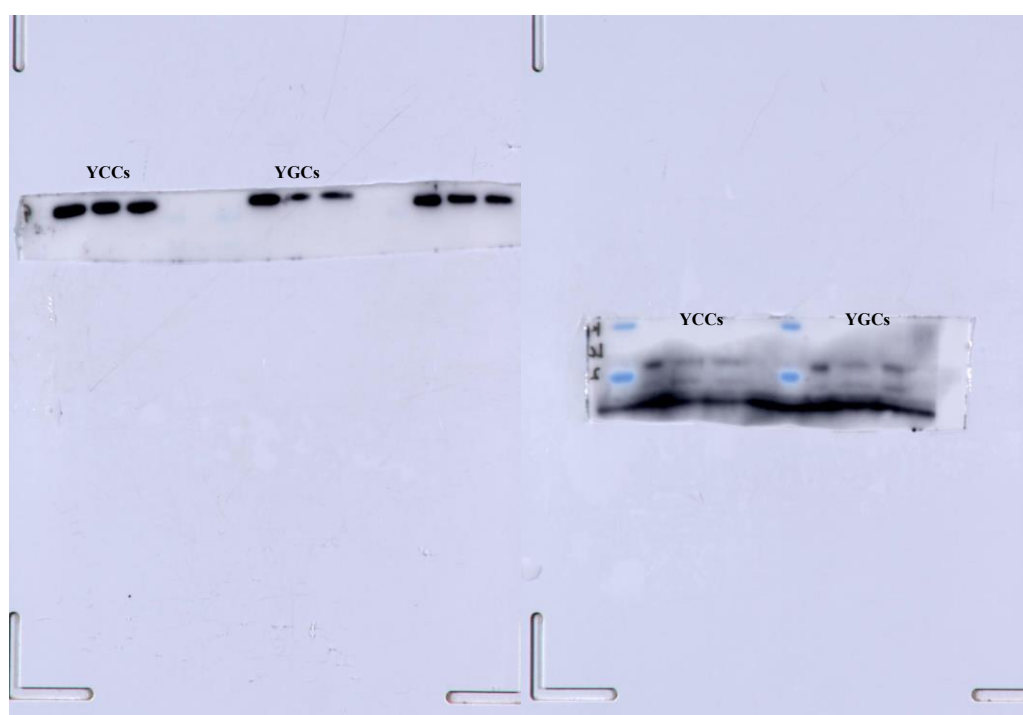

GAPDH

LC3B

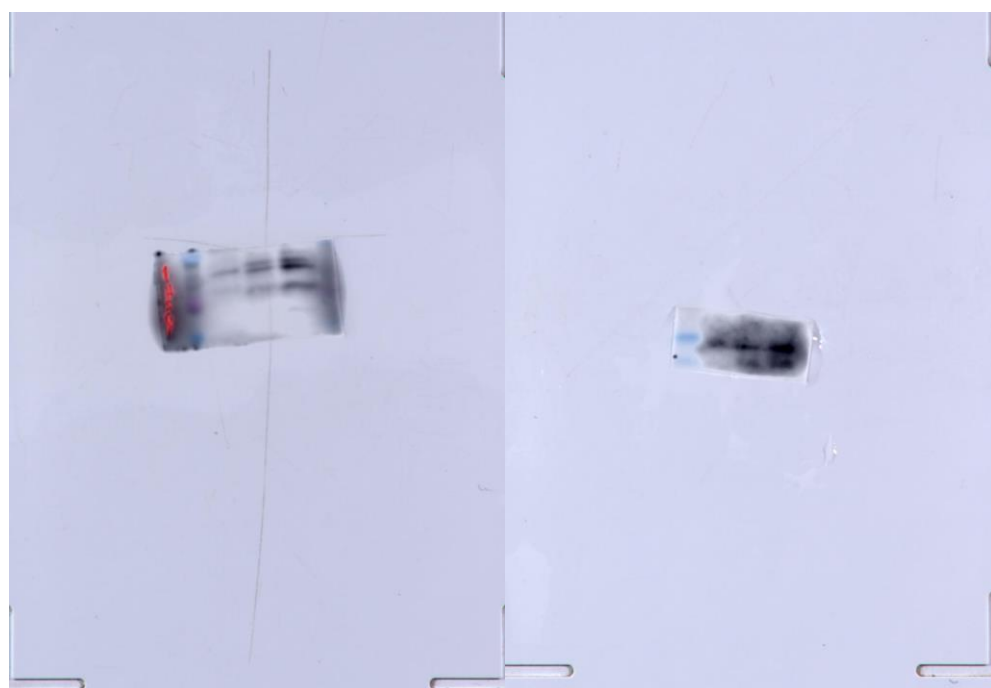

BNIP3

CIRBP

Supplement: Supplementary file 1 [file biomolecules-15-00759-s001.zip › FIG 1 C.pdf]

FIG2

FIG2 A

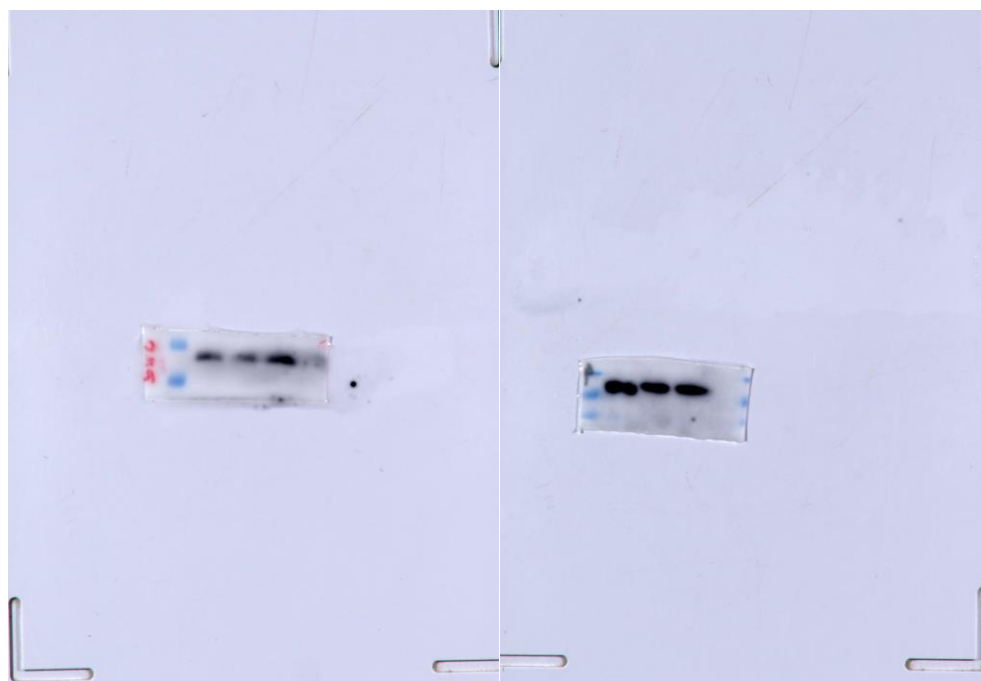

CIRBP

β-actin

FIG2 C

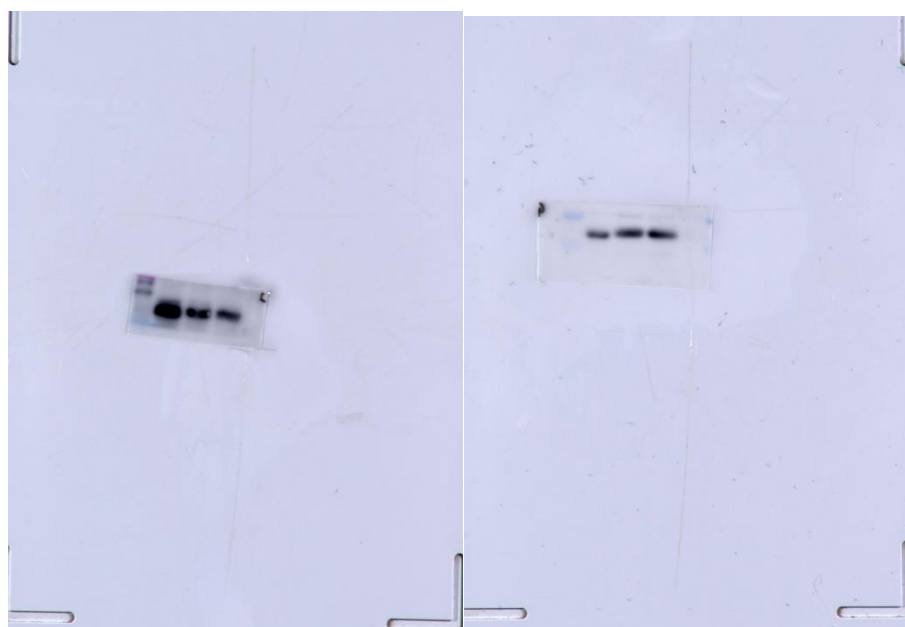

P62

ATG5

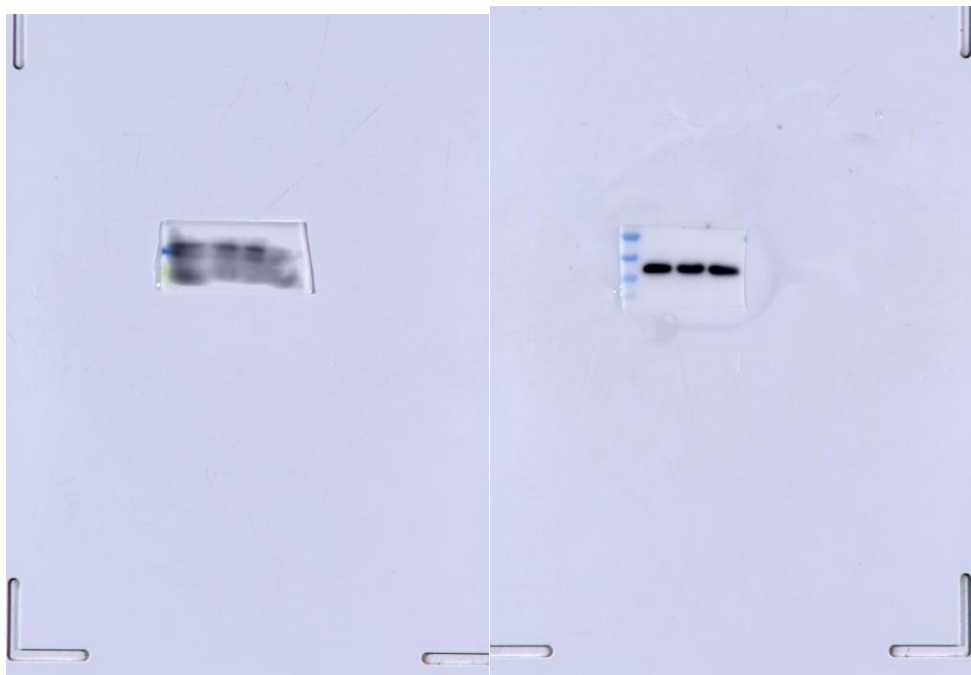

LC3B

GAPDH

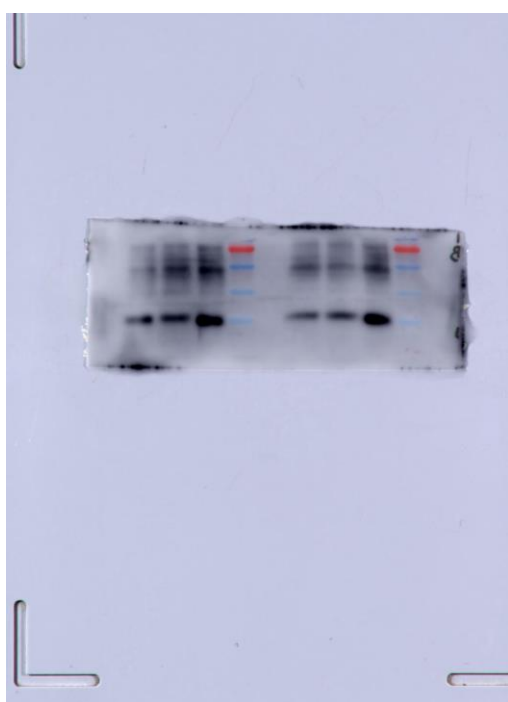

Beclin-1

FIG2 D

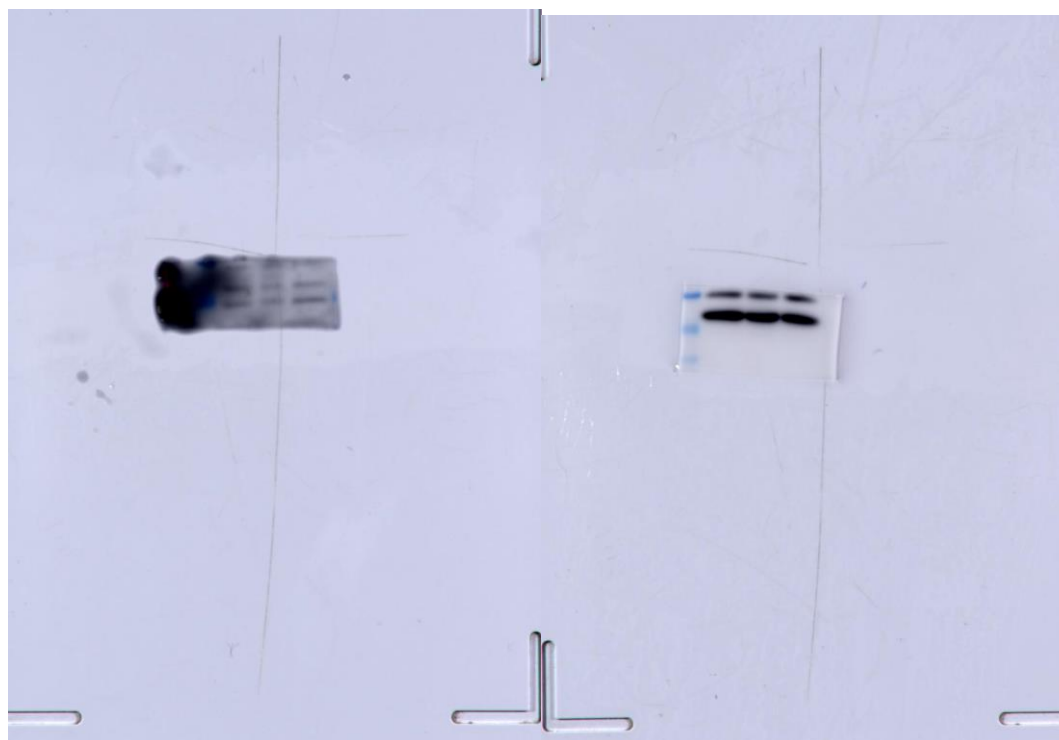

BNIP3

GAPDH

Supplement: Supplementary file 1 [file biomolecules-15-00759-s001.zip › FIG2.pdf]

**FIG3**

**FIG3 D**

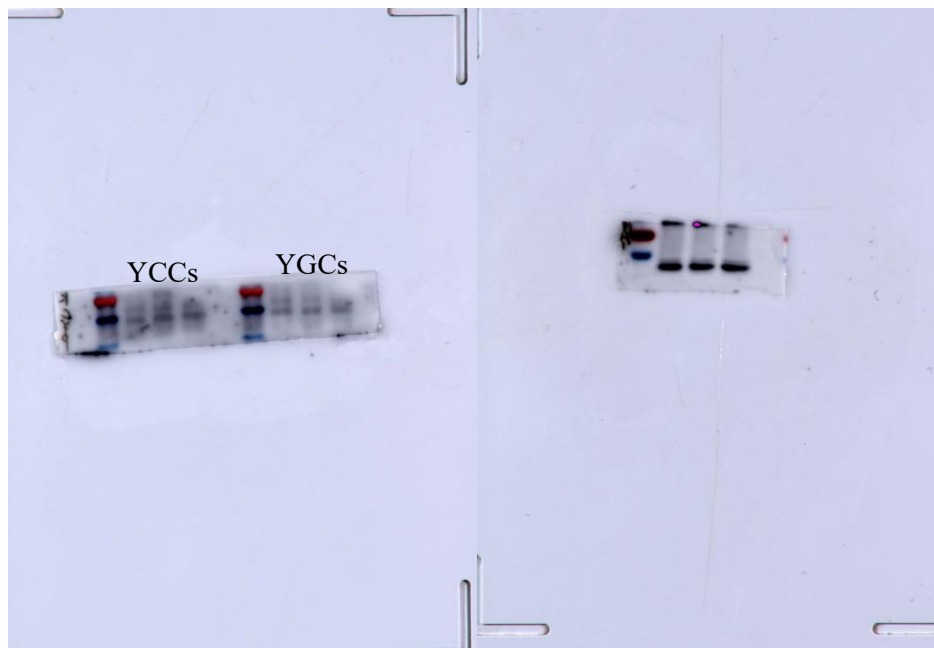

**CYP17A1**

**STAR**

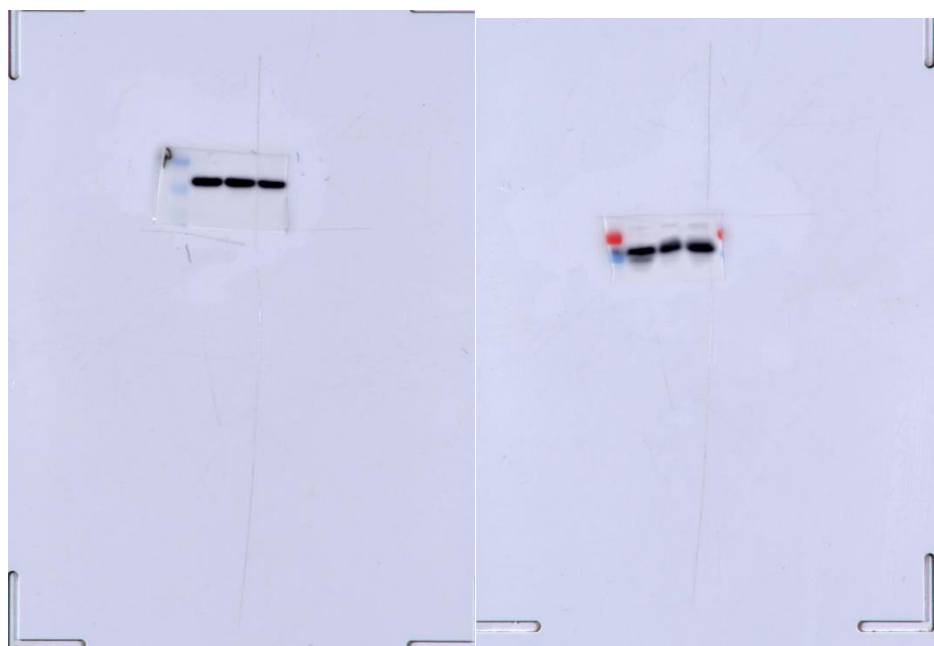

**GAPDH**

**CYP19A1**

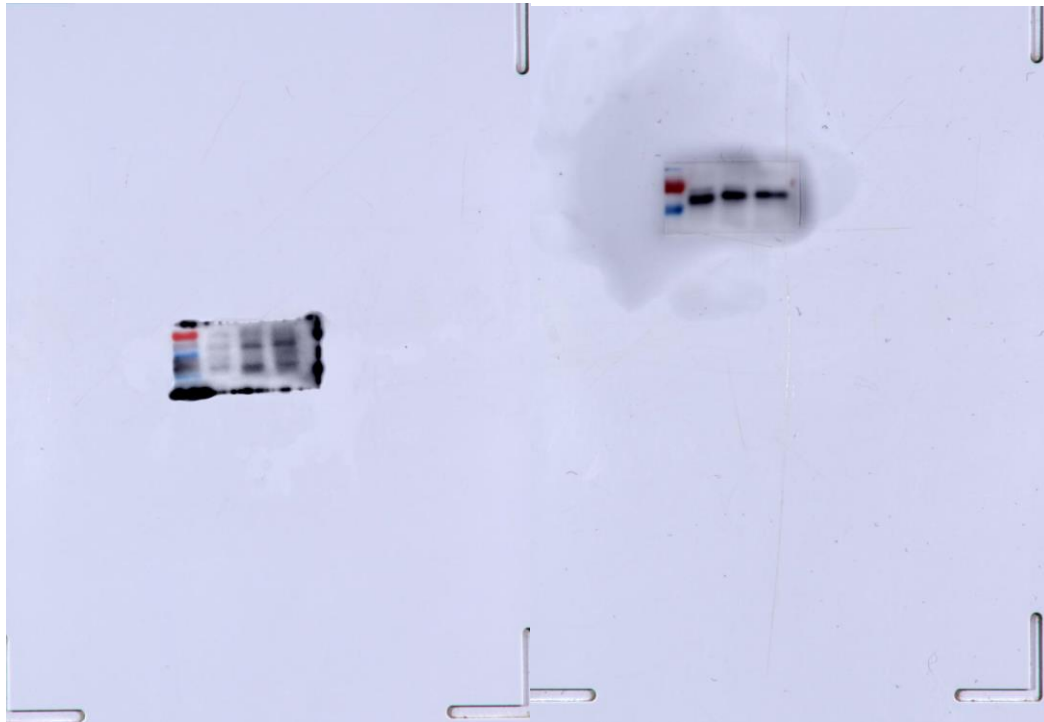

CYP1B1

CYP1A1

**FIG3 E**

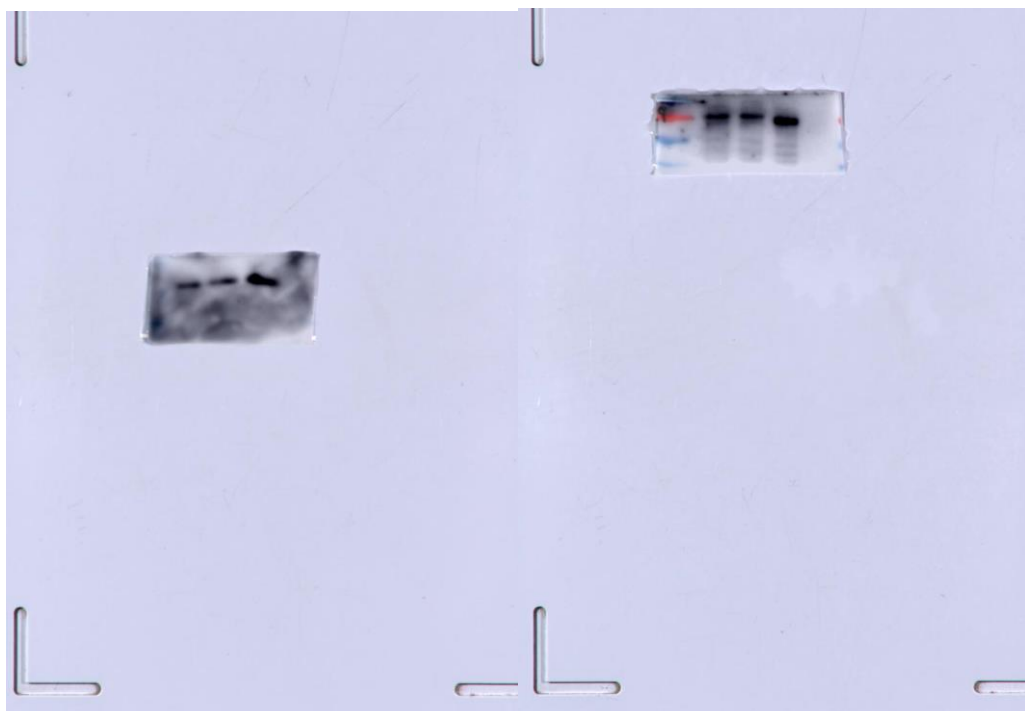

TSG6

COX2

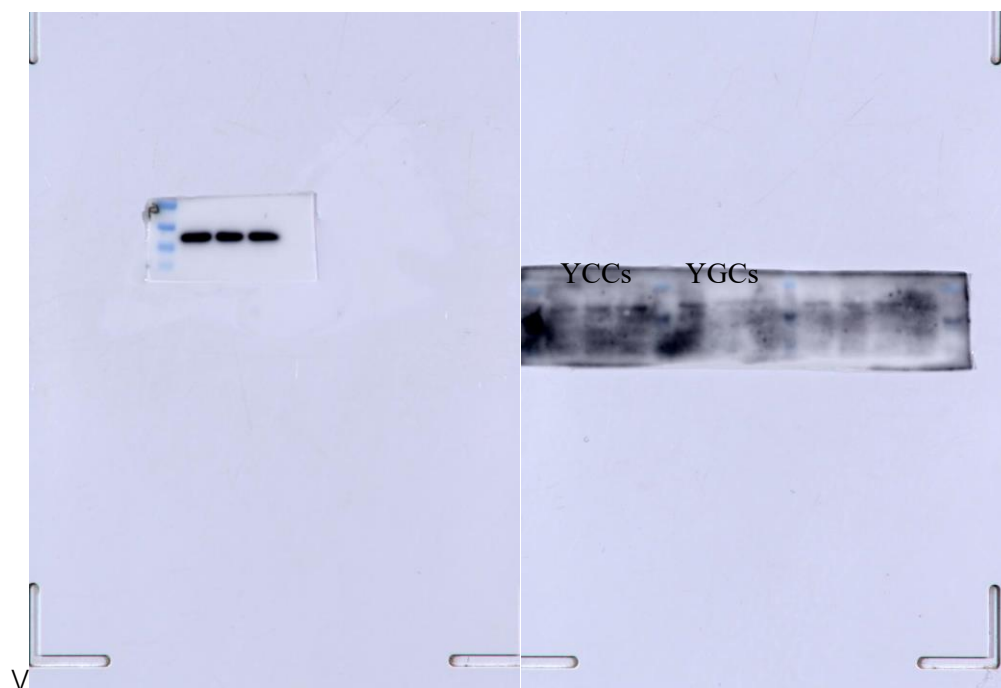

GAPDH

PTX3

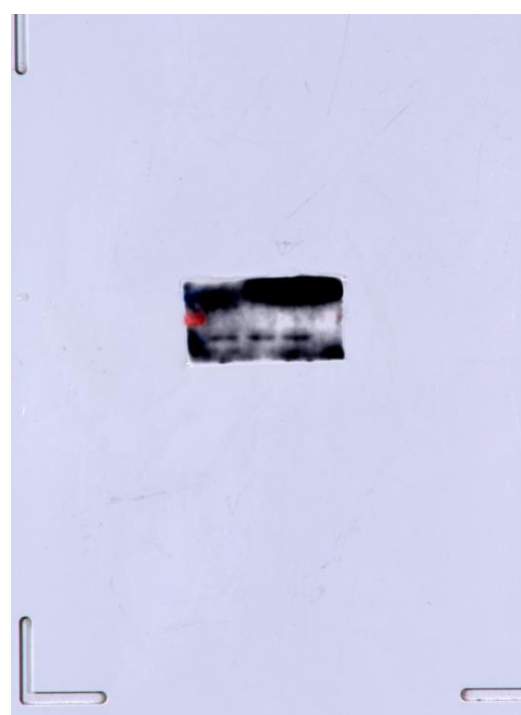

HAS2

Supplement: Supplementary file 1 [file biomolecules-15-00759-s001.zip › FIG3.pdf]

**FIG4 D**

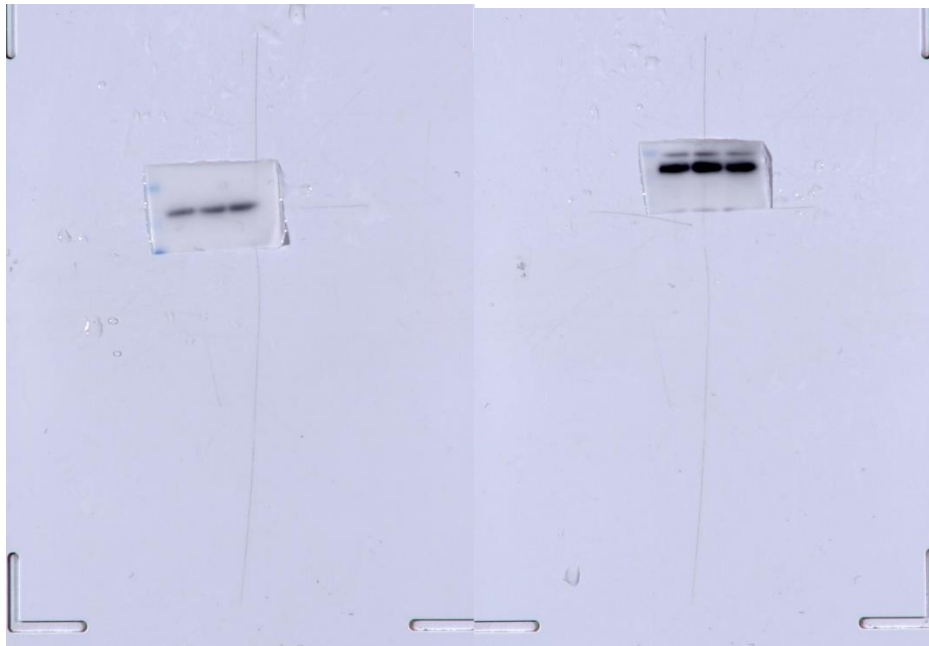

**BCL-2**

**GAPDH**

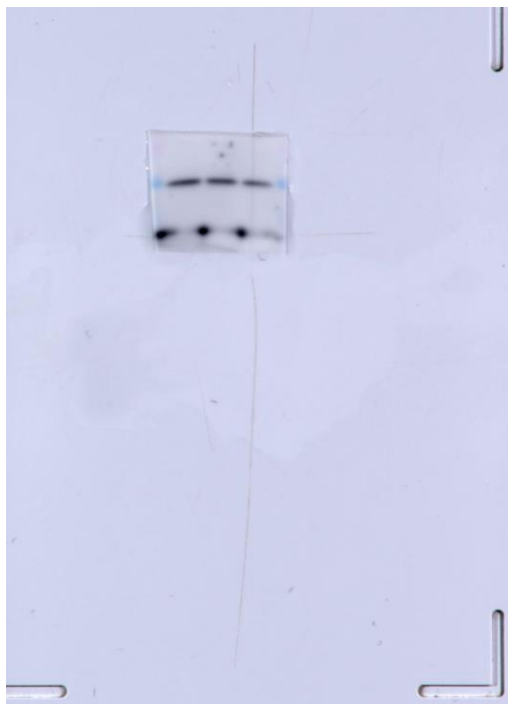

**BAX**

Supplement: Supplementary file 1 [file biomolecules-15-00759-s001.zip › FIG4 D.pdf]

FIG5

FIG 5A

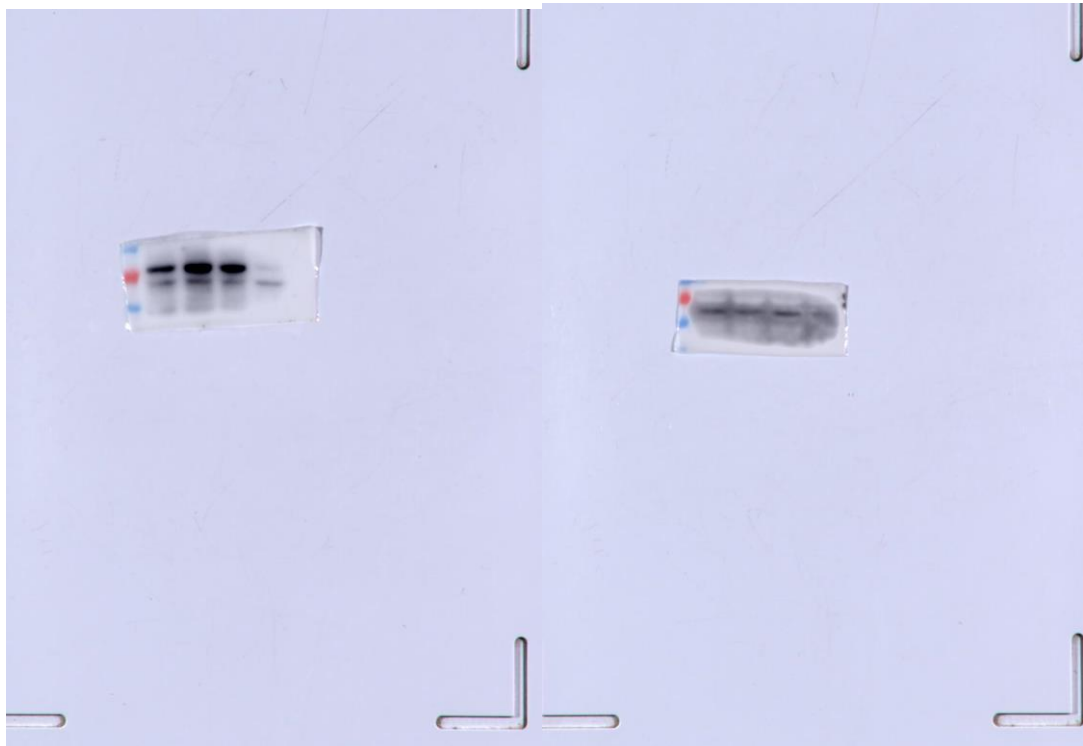

P62

Beclin-1

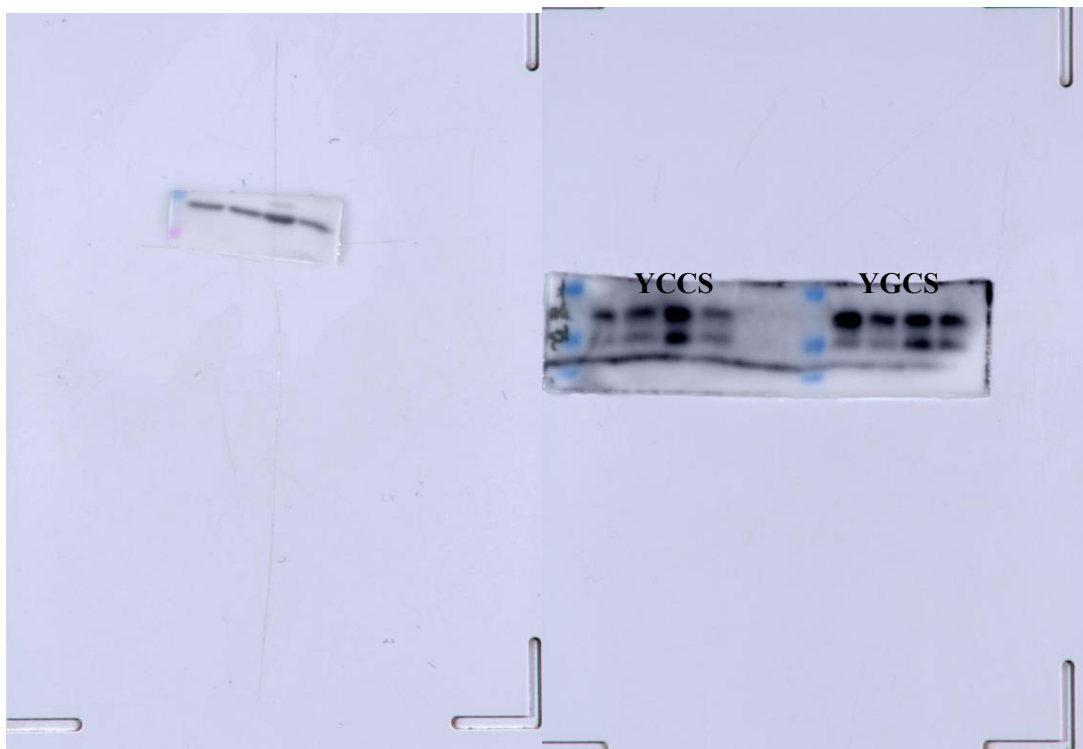

ATG5

Ic3B

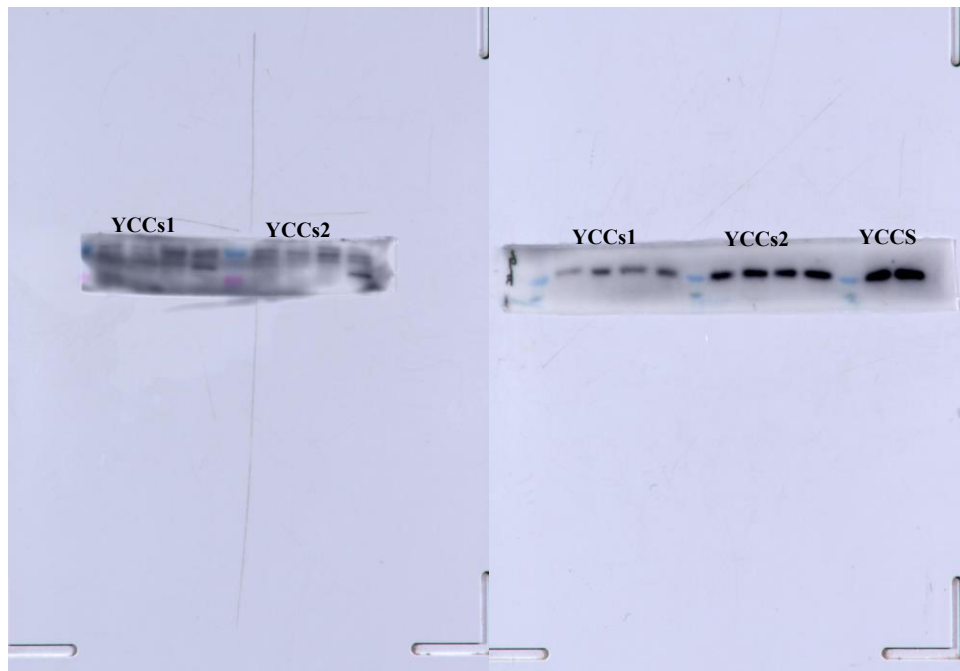

BNIP3

GAPDH

FIG5 C

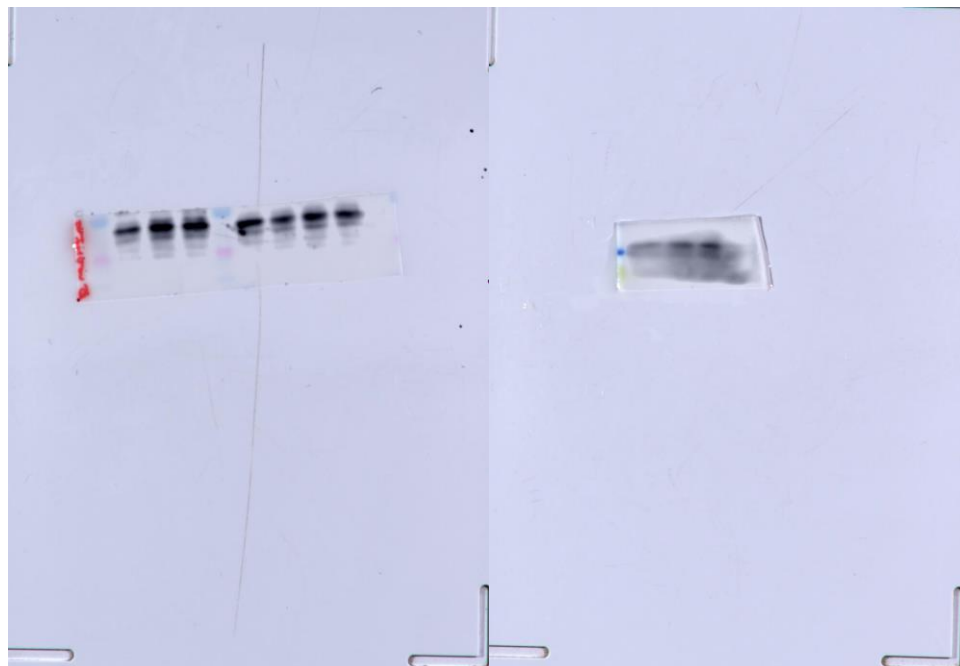

BNIP3

LC3B

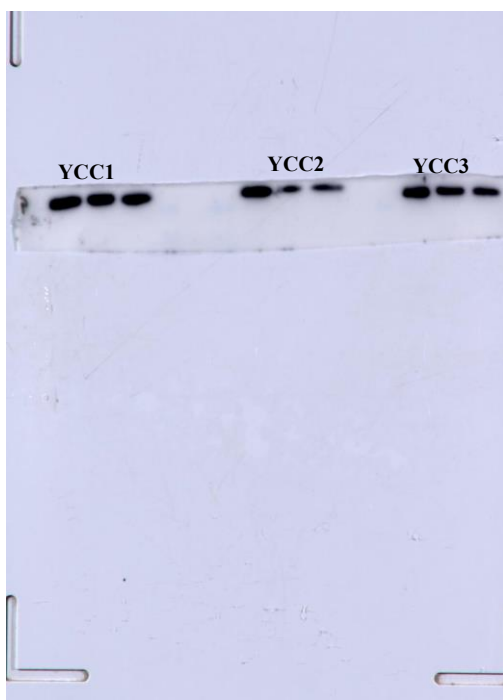

GAPDH

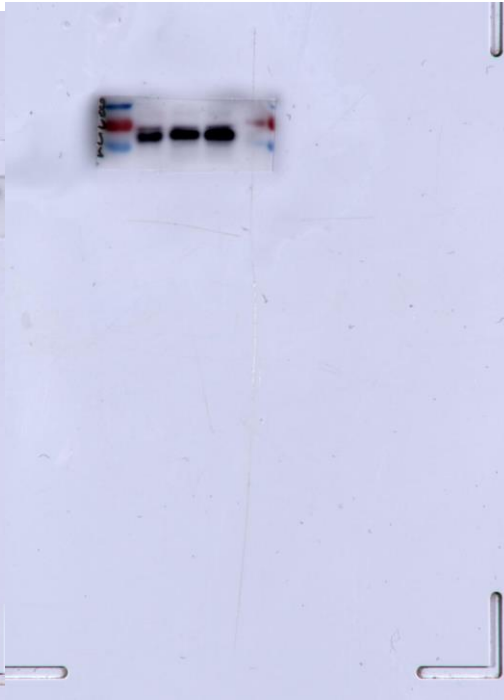

Beclin-1

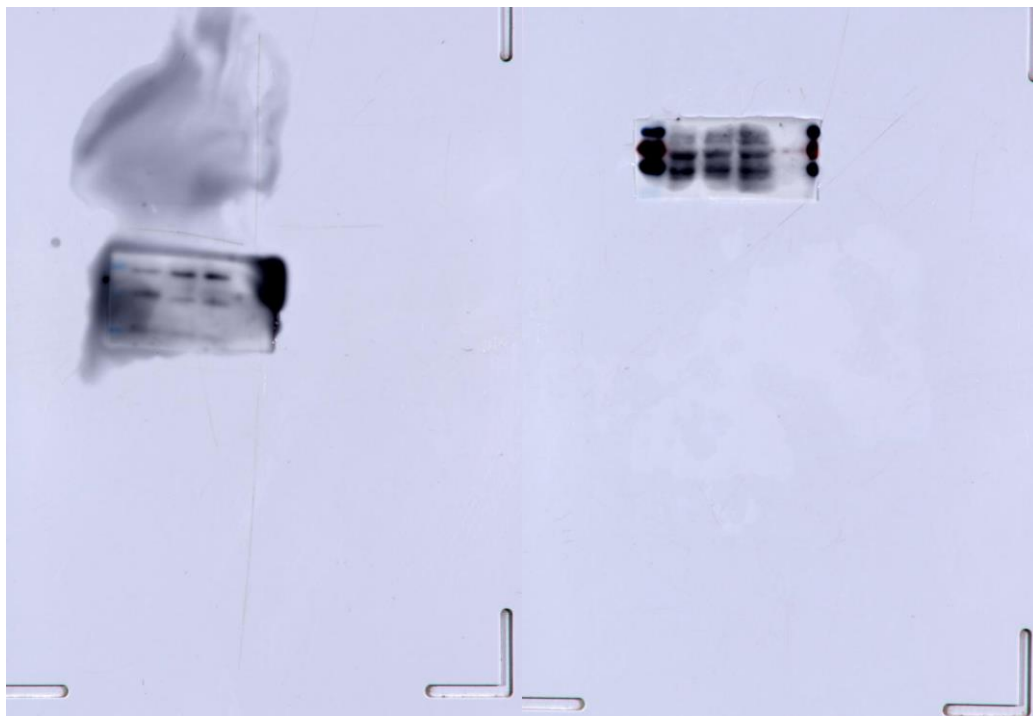

ATG5

P62

Supplement: Supplementary file 1 [file biomolecules-15-00759-s001.zip › FIG5.pdf]

**FIG8**

**FIG8 A**

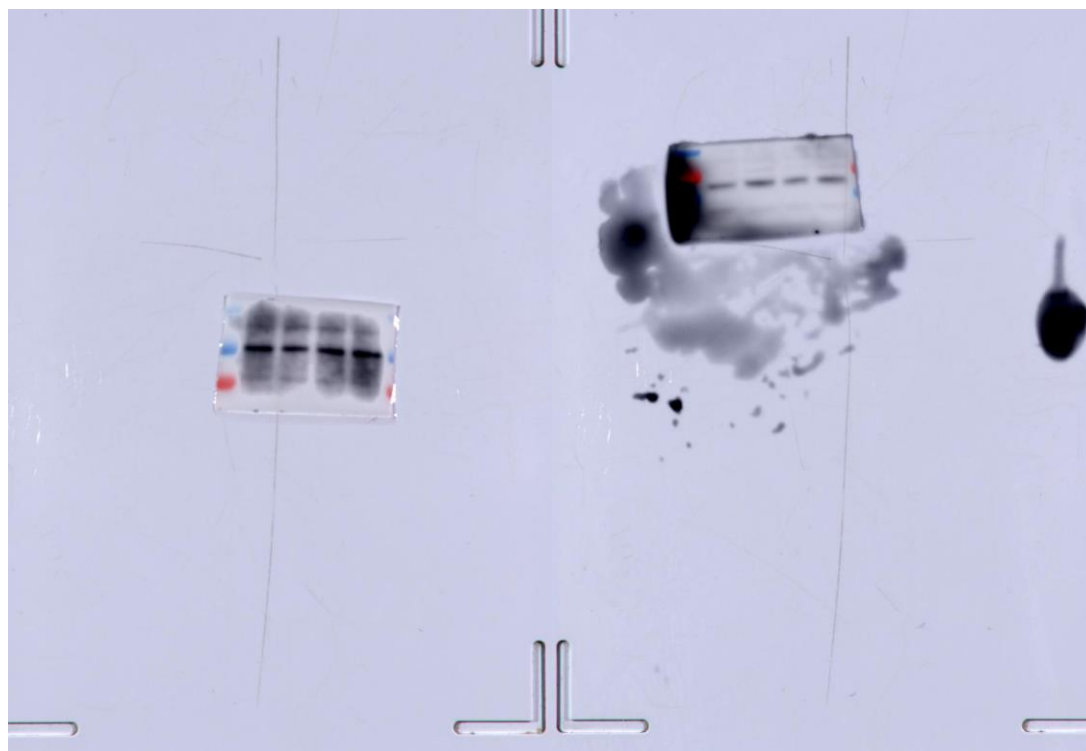

**PI3K**

**P-AKT**

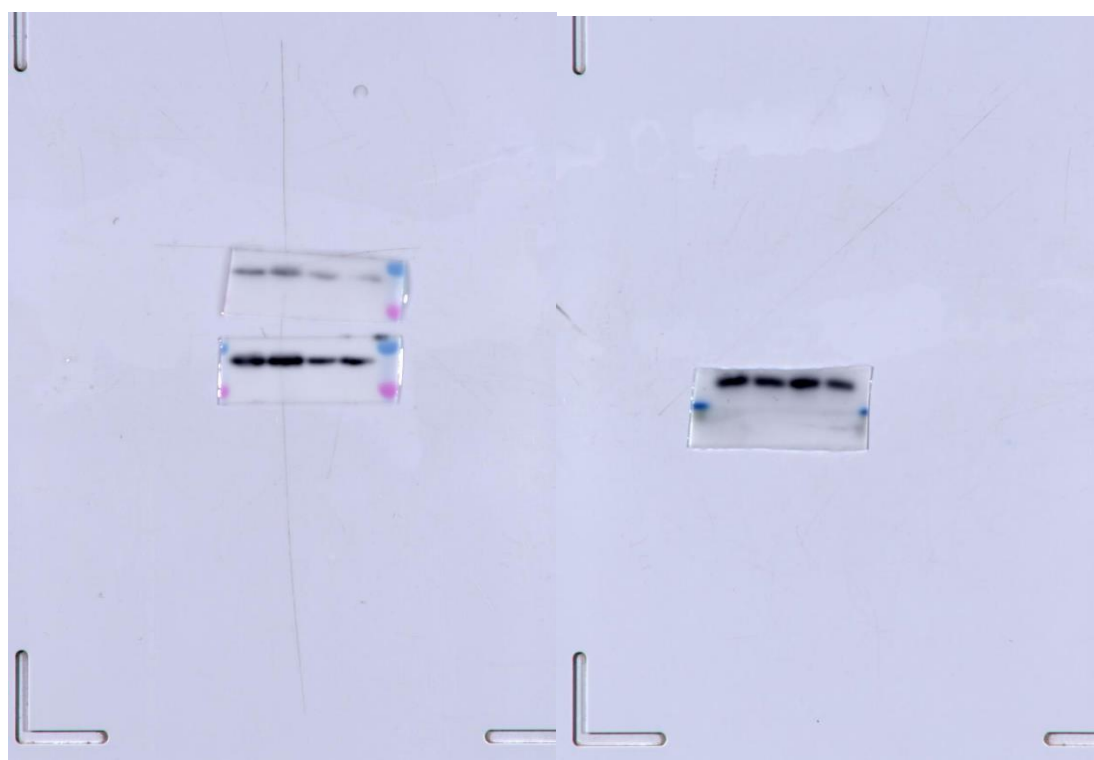

**BCL-2**

**BAX**

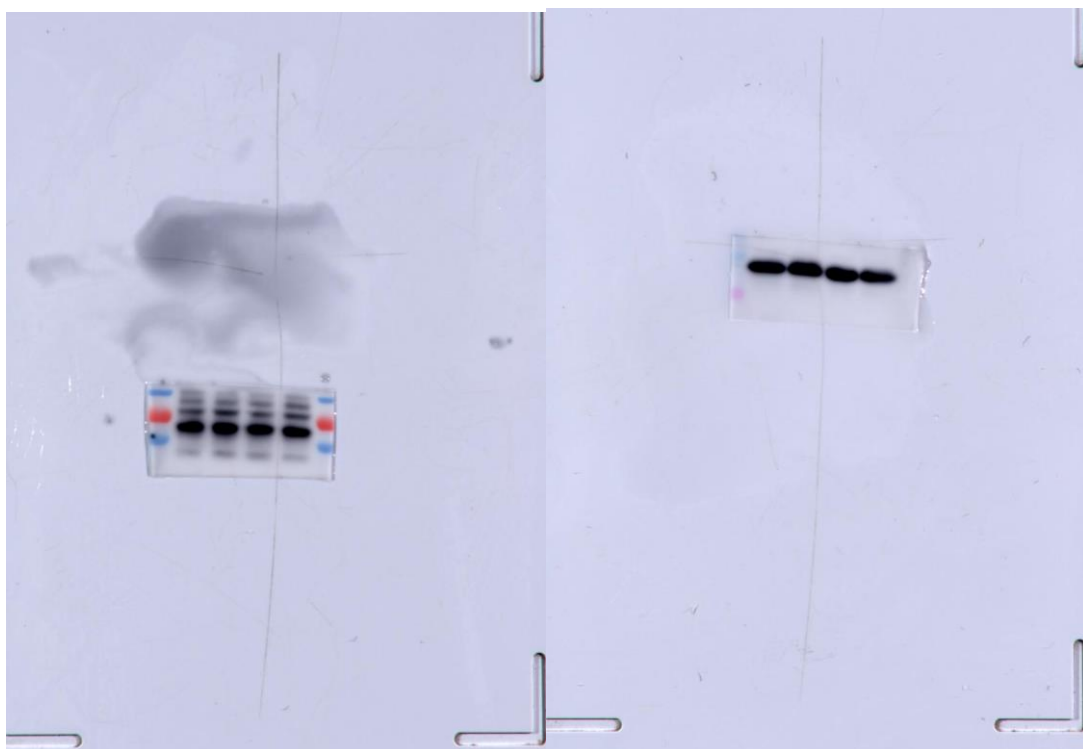

**AKT**

**GAPDH**

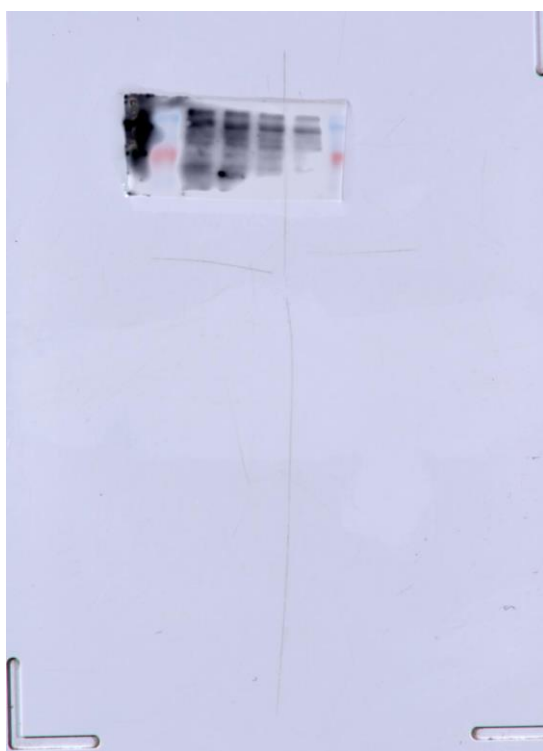

**P-PI3K**

**FIG8 B**

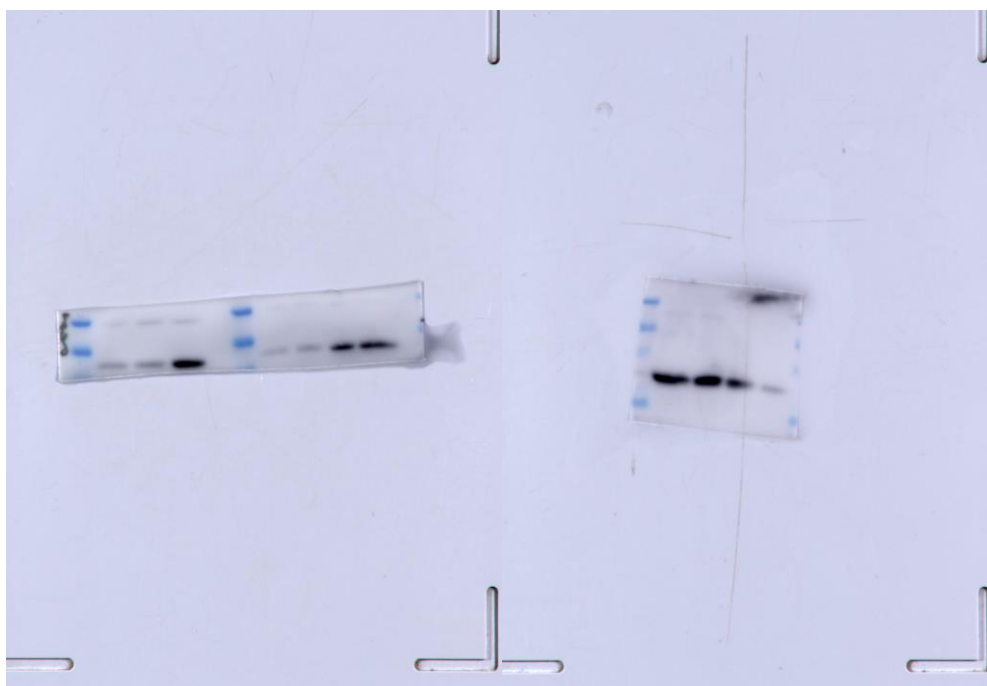

**BCL-2**

**BAX**

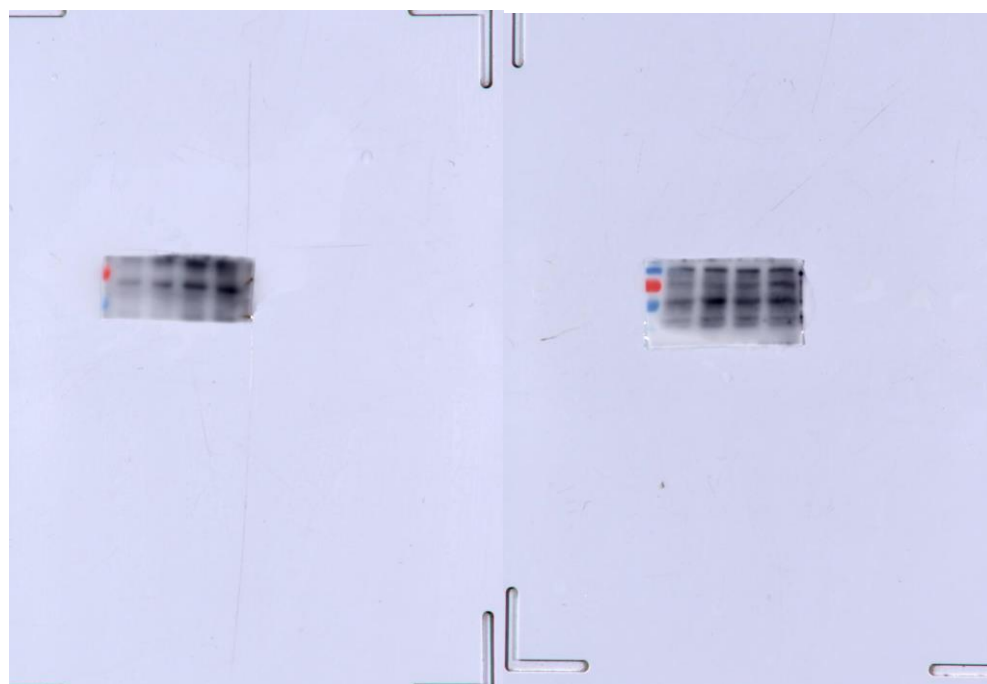

**P-AKT**

**P-PI3K**

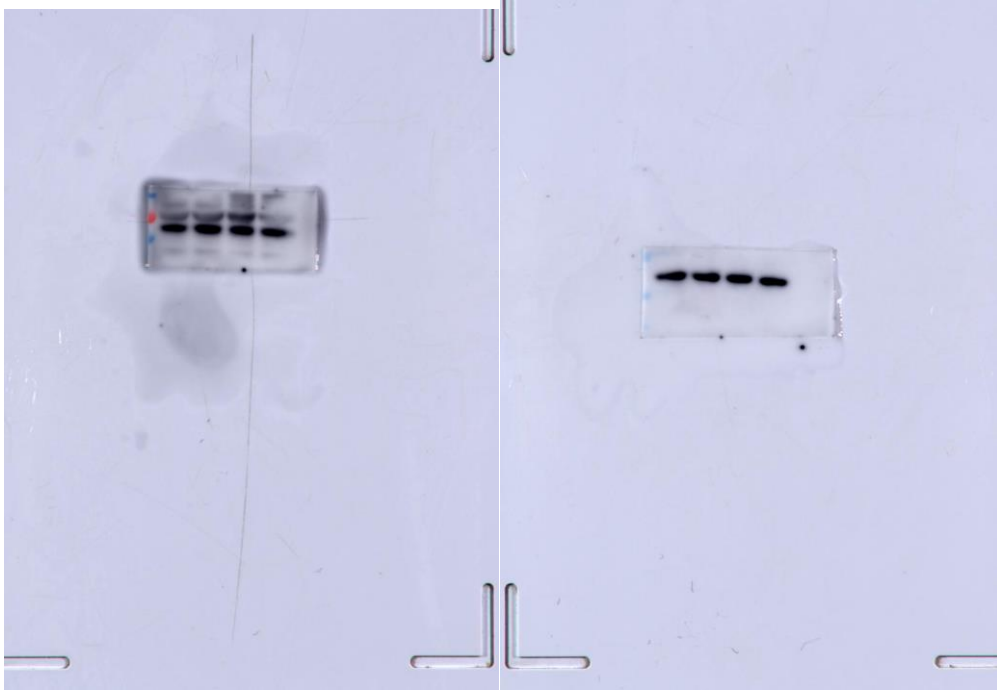

**AKT**

**GAPDH**

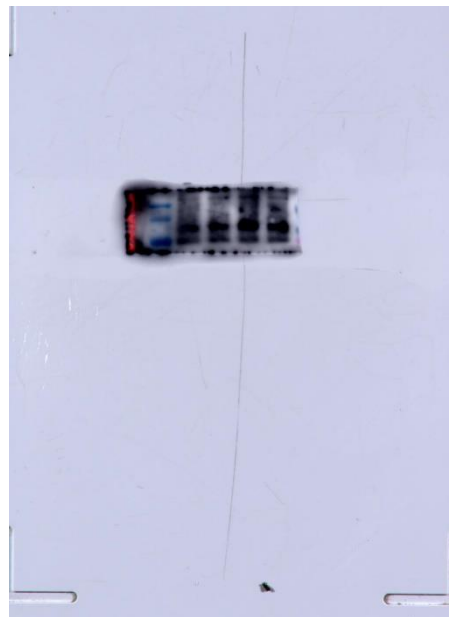

**P-PI3K**

Supplement: Supplementary file 1 [file biomolecules-15-00759-s001.zip › FIG8.pdf]
